# Genome-Wide Comprehensive Identification and *In Silico* Characterization of Lectin Receptor-Like Kinase Gene Family in Barley (*Hordeum vulgare* L.)

**DOI:** 10.1155/2024/2924953

**Published:** 2024-02-27

**Authors:** Fee Faysal Ahmed, Farah Sumaiya Dola, Md Shohel Ul Islam, Fatema Tuz Zohra, Nasrin Akter, Shaikh Mizanur Rahman, Md. Abdur Rauf Sarkar

**Affiliations:** ^1^Department of Mathematics, Faculty of Science, Jashore University of Science and Technology, Jashore 7408, Bangladesh; ^2^Department of Genetic Engineering and Biotechnology, Faculty of Biological Science and Technology, Jashore University of Science and Technology, Jashore 7408, Bangladesh; ^3^Department of Genetic Engineering and Biotechnology, Faculty of Biological Sciences, University of Rajshahi, Rajshahi 6205, Bangladesh

## Abstract

Lectin receptor-like kinases (LecRLKs) are a significant subgroup of the receptor-like kinases (RLKs) protein family. They play crucial roles in plant growth, development, immune responses, signal transduction, and stress tolerance. However, the genome-wide identification and characterization of *LecRLK* genes and their regulatory elements have not been explored in a major cereal crop, barley (*Hordeum vulgare* L.). Therefore, in this study, integrated bioinformatics tools were used to identify and characterize the LecRLK gene family in barley. Based on the phylogenetic tree and domain organization, a total of 113 *LecRLK* genes were identified in the barley genome (referred to as *HvlecRLK*) corresponding to the *LecRLK* genes of *Arabidopsis thaliana*. These putative *HvlecRLK* genes were classified into three groups: 62 G-type *LecRLKs*, 1 C-type *LecRLK*, and 50 L-type *LecRLKs*. They were unevenly distributed across eight chromosomes, including one unknown chromosome, and were predominantly located in the plasma membrane (G-type *HvlecRLK* (96.8%), C-type *HvlecRLK* (100%), and L-type *HvlecRLK* (98%)). An analysis of motif composition and exon-intron configuration revealed remarkable homogeneity with the members of *AtlecRLK*. Notably, most of the *HvlecRLKs* (27 G-type, 43 L-type) have no intron, suggesting their rapid functionality. The Ka/Ks and syntenic analysis demonstrated that *HvlecRLK* gene pairs evolved through purifying selection and gene duplication was the major factor for the expansion of the HvlecRLK gene family. Exploration of gene ontology (GO) enrichment indicated that the identified *HvlecRLK* genes are associated with various cellular processes, metabolic pathways, defense mechanisms, kinase activity, catalytic activity, ion binding, and other essential pathways. The regulatory network analysis identified 29 transcription factor families (TFFs), with seven major TFFs including bZIP, C2H2, ERF, MIKC_MADS, MYB, NAC, and WRKY participating in the regulation of *HvlecRLK* gene functions. Most notably, eight TFFs were found to be linked to the promoter region of both L-type *HvleckRLK64* and *HvleckRLK86*. The promoter cis-acting regulatory element (CARE) analysis of barley identified a total of 75 CARE motifs responsive to light responsiveness (LR), tissue-specific (TS), hormone responsiveness (HR), and stress responsiveness (SR). The maximum number of CAREs was identified in *HvleckRLK11* (25 for LR), *HvleckRLK69* (17 for TS), and *HvleckRLK80* (12 for HR). Additionally, *HvleckRLK14, HvleckRLK16, HvleckRLK33, HvleckRLK50, HvleckRLK52, HvleckRLK56, and HvleckRLK110* were predicted to exhibit higher responses in stress conditions. In addition, 46 putative miRNAs were predicted to target 81 *HvlecRLK* genes and *HvlecRLK13* was the most targeted gene by 8 different miRNAs. Protein-protein interaction analysis demonstrated higher functional similarities of 63 HvlecRLKs with 7 *Arabidopsis* STRING proteins. Our overall findings provide valuable information on the LecRLK gene family which might pave the way to advanced research on the functional mechanism of the candidate genes as well as to develop new barley cultivars in breeding programs.

## 1. Introduction

The physiological developments of plants face constant threats from pathogenic organisms and environmental stresses. Plants have evolved mechanisms to identify pathogens through cell-surface receptors which contribute to their innate immunity and protect themselves from invading pathogens [1, 2]. Pattern recognition receptors (PRRs) are a crucial component of plant immunity, localized in the cell membrane where they serve as the first line of defense by initiating early immune response [3]. PRRs form complexes with other molecules, allowing them to recognize microbial molecules like pathogen/microbe-associated molecular patterns (PAMPs/MAMPs) or damage-associated molecular patterns (DAMPs), initiating signal transduction cascades [4–7]. As a result, PRRs play a pivotal role in sensing PAMPs and triggering immune responses. Plant PRRs can be categorized into two main types: receptor-like kinases (RLKs), which possess an intracellular kinase domain, and receptor-like proteins (RLPs), which lack a known intracellular signaling domain [4].

The interaction between plants and various environmental conditions involves numerous signal recognition and transduction pathways, including the RLK superfamily, a large group of cell-surface receptors dominantly localized in the cell membrane [8]. RLKs play a vital role in receiving and transmitting numerous signals and regulating various activities, such as disease resistance, self-incompatibility, hormonal sensing, and plant development [9, 10]. Typically, RLKs consist of three main parts: an extracellular N-terminal ligand-binding domain for signal reception, an intermediate transmembrane region for anchoring the protein in the membrane, and an intracellular C-terminal kinase domain responsible for initiating plant immunity [8, 10, 11]. RLKs can be classified into 17 subgroups based on the variability of the extracellular domain [12, 13]. In higher plants, these receptors were first identified in maize, and subsequently, numerous RLKs were found in over 20 plant species [14].

Lectin receptor-like kinases (LecRLKs) are characterized by the presence of an extracellular lectin domain at the N-terminus [15, 16]. The diverse lectin domain at the N-terminus allows lecRLKs to recognize environmental stimuli, while the intracellular kinase domain at the C-terminus phosphorylates downstream proteins to transmit signals [15, 17]. Depending on the type of lectin domain, LecRLKs are further classified into 3 subfamilies: (i) L-type, (ii) G-type, and (iii) C-type LecRLK [10]. The L-type (legume-like) LecRLKs are identified by their lectin-legB domain and/or a protein kinase domain, mainly found in legumes [18–20]. Despite having a *β*-sandwich fold structure, these proteins are soluble and exhibit glucose/mannose-binding affinity. L-type LecRLKs are found on cell membranes and have a conserved hydrophobic cavity for binding with hydrophobic ligands [21]. Additionally, they play an important role in various physiological functions, including pollen development and pathogen resistance [22–24]. G-type LecRLKs are mainly *Galanthus nivalis*agglutinin-related lectins which were previously named B-type LecRLKs as they have similarities in their extracellular domains with bulb lectin proteins. Having an S-locus region participating in self-incompatibility reactions, G-type LecRLKs are also known as S-domain RLKs [20, 25, 26]. Many G-type LecRLKs contain a plasminogen apple nematode (PAN) domain and an epidermal growth factor (EGF) domain [27]. The EGF motif is cysteine-rich, likely contributing to the formation of disulfide bonds, while the PAN motif is associated with protein-protein and protein-carbohydrate interactions [28]. G-type LecRLKs, such as Pi-d2 in rice, have been shown to confer resistance to the fungus *Magnaporthe grisea* [29] and also exhibit resistance against dark-induced leaf senescence, bacteria, and insects [30–32]. C-type LecRLKs are a subfamily of calcium-dependent RLKs which are predominantly found in mammals rather than plants [33]. This subfamily is the smallest among plant LecRLKs, with only a single C-type lectin protein identified in the genomes of rice and *Arabidopsis* (*Arabidopsis thaliana*) [27] and two in soybean (*Glycine max*) [34] and wheat (*Triticum aestivum*) [35]. Although L-type and G-type lectin kinases are plant-specific [10, 22, 36], C-type lectin kinases have been identified in *Hydra vulgaris* where they are involved in immune response [37].

Despite being abundant in plants, research on the biological roles of *LecRLKs* is limited [20, 38]. Previous research has identified 75 *LecRLK* genes in *Arabidopsis* (*A. thaliana*) [27], 173 in rice (*Oryza sativa*) [27], 231 in *Populous* (*Populus trichocarpa*) [39], 185 in soybean (*G. max*) [34], 263 in wheat (*T. aestivum*) [35], 22 in tomato (*Solanum lycopersicum*) [40], 113 in potato (*Solanum tuberosum*) [41], and 46 in cucumber (*Cucumis sativus* L.) [42]. *LecRLKs* play a pivotal role in plant growth, stress management, and innate immune responses [23, 43, 44]. For instance, in *Arabidopsis* (*A. thaliana*) *LecRK-b2*, an L-type receptor-like kinas is induced by salinity, osmotic stress, and abscisic acid [45]. Another L-type receptor-like kinase, *LECRK-IV.2*, plays a crucial role in *Arabidopsis* pollen sterility. Mutation of *LECRK-IV.2* is responsible for the deformation of pollen grain in *Arabidopsis* [22]. In rice (*O. sativa*), the *OslecRK* maintains seed viability via modulating the expression pattern of *α*-amylase genes. Mutations in *OslecRK* reduce the plant resistance to microbes and herbivorous insects [46]. *LecRLKs* are implicated in senescence and wounding stress responses, plant legume-rhizobium symbiotic relationships, fiber growth in cotton plants, and pollen development. Furthermore, they are known to exhibit hypersensitivity responses during pathogen attack and confer resistance against fungal pathogens, perceive insect feeding, and provide salt tolerance responses [29, 38, 44, 47–50].

Barley (*H*. *vulgare* L.) is a diploid plant with 14 chromosomes and a large genome of 5.1 gigabases (Gb). It is one of the oldest domesticated cereal crops globally and holds significant economic value. Generally, barely is commonly used for human diets, livestock feed, and as a raw material in the malting and brewing industries [51, 52]. It ranked as the fourth most abundant cereal crop in terms of cultivated area and yield (FAO: https://faosta.fao.org). Additionally, barley is one of the most stress-resistant crops, such as salt, cold, and soil infertility stress, having modulated genetic sequence organizations against biotic and abiotic stress [53].

Bioinformatics analysis tools have significantly promoted the identification and *in silico* characterization of genes which have been developing new features day by day. Nevertheless, few bioinformatics analyses were reported on *LecRLKs* in various plant species, and no genome-wide identification and functional analysis of *LecRLKs* have been carried out in *H. vulgare*, a major economically important crop species. In this study, we comprehensively identified *LecRLK* genes in barley (*H. vulgare*) across the genome using integrated bioinformatics approaches. We further analyzed their phylogenetic relationships, gene structures, conserved domain, motifs, chromosomal distribution, subcellular localization, gene ontology, transcription factors, and cis-regulatory elements in the promoter region. This study will serve as a foundational resource for in-depth studies on the functions and responses of *LecRLK*s to environmental stresses.

## 2. Materials and Methods

### 2.1. Database Search and Retrieval of Lectin Receptor-Like Kinase (LecRLK) Protein Sequences in Barley Genome

The complete genome data and protein sequences of *H. vulgare* were obtained from Phytozome v13.0 (https://phytozome-next.jgi.doe.gov/) (S1 Data) [54]. To identify all members of the LecRLK protein family in the *H. vulgare* genome, we utilized the LecRLK protein sequence and annotation information from *Arabidopsis* (*A. thaliana*), available in the TAIR database (https://www.arabidopsis.org/). Protein domains including Lectin_legB (PF00139), Pkinase (PF00069), PK_Tyr_Ser-Thr (PF07714), Lectin_C (PF00059), B_lectin (PF01453), and S_locus_glycop (PF01453) of the LecRLK family were obtained from the Pfam database (https://pfam.janelia.org/) using the Hidden Markov Model (HMM) profile. Subsequently, the possible candidate LecRLK protein sequence in *H. vulgare* was retrieved through Pfam (https://pfam.xfam.org/family) [55], NCBI-CDD (https://www.ncbi.nlm.nih.gov/cdd/) [56], and SMART (https://smart.embl-heidelberg.de/) [57] online tools to predict protein conserved domains and was used for further analysis.

### 2.2. Determination of Physiochemical Properties of Barley *LecRLK* Genes

The primary transcript, gene length, chromosomal location, and open reading frame (ORF) of the identified *LecRLK* genes were retrieved from the *H. vulgare* genome database in Phytozome. Furthermore, the basic physiochemical properties of proteins encoded by the *LecRLK* gene in barely, including length, molecular weight, and isoelectric points (pI), of predicted proteins, were analyzed by the online tools ExPASy (https://web.expasy.org/protparam/) [58].

### 2.3. Phylogenetic Relationship of LecRLK Proteins in Barley and *Arabidopsis*

The protein sequences encoded by the *LecRLK* gene in barely (*H. vulgare*) and *Arabidopsis* (*A. thaliana*) retrieved from Phytozome v13 (https://phytozome.jgi.doe.gov/pz/portal.html/) were used to conduct the phylogenetic tree analysis. We imported all LecRLK protein sequences using MEGA 11.0 software [59] and performed multiple sequence alignments using the Clustal-W method [60] with the default parameters and 1000 bootstrap values. Finally, the phylogenetic tree was constructed using the neighbor-joining method [61] and evolutionary distances were calculated using the Equal Input method [62]. The constructed phylogenetic tree was then presented using iTOL v6.74 (https://itol.embl.de/) [63].

### 2.4. Conserved Domain and Motif Analysis of LecRLK Proteins in Barley

We analyzed the conserved domains of identified barely (*H. vulgare*) LecRLK proteins in comparison to *Arabidopsis* (*A. thaliana*) LecRLK proteins based on Pfam [64], SMART [57], and NCBI-CDD [56] online databases. Moreover, we predicted the similarity and dissimilarity of structural motifs in barley (*H. vulgare*) and *Arabidopsis* (*A. thaliana*) proteins using the Multiple Expectation Maximization for Motif Elicitation (https://meme-suite.org/meme/tools/meme) (https://meme.nbcr.net/meme/) tools of MEME-Suite (https://meme-suite.org/meme/) [65]. The MEME analysis was performed with specific parameters including an optimum motif width of ≥6 and ≤50 and a maximum motif number of 20.

### 2.5. Gene Structure Analysis of *LecRLKs* in Barley

To analyze the gene structure including exon-intron organization of predicted *HvLecRLKs*, CDS and genomic DNA sequences in FASTA format were obtained from Phytozome v13 (S2 Data and S3 Data). The predicted *HvLecRLK* gene structure was analyzed by an online software program Gene Structure Display Server GSDS2.0 (https://gsds.cbi.pku.edu.cn/) [66] based on the DNA sequences of identified *LecRLK* genes compared to the *Arabidopsis LecRLK* genes.

### 2.6. Gene Duplication Analysis and Synonymous (Ks) and Nonsynonymous (Ka) Substitution Ratio Calculation

The synonymous (Ks) and nonsynonymous (Ka) substitution ratios of barley *lecRLK* were illustrated using TBtools version-v1.116 [67]. Furthermore, molecular evolution was estimated using Ka/Ks ratios of paralogous gene pairs. Moreover, we calculated the duplication and divergence period (in millions of years ago) using a synonymous mutation rate of substitutions per synonymous site per year as T = Ks/2*λ* (*λ* = 6.5 × 10^−9^) × 10^−6^ [68].

### 2.7. Collinearity and Synteny Analysis of the LecRLK Gene Family of Barley

The Plant Genome Duplication Database (https://chibba.agtec.uga.edu/duplication/index/locus) was used to confirm the gene duplication in barley and *Arabidopsis lecRLK* genes. Furthermore, TBtools version-v1.116 was used to illustrate the collinear and syntenic gene pairs of the *HvlecRLK* and *AtlecRLK* gene families [67].

### 2.8. Analysis of Chromosomal Location of *LecRLK* Genes in Barley

To predict the chromosomal location of *HvLecRLKs*, the barley (*H. vulgare*) genomic information was retrieved from the Phytozome v13 database. Chromosomal locations of the *LecRLK* genes of barely were determined using the tools MapGene2Chromosome V2 web server (https://mg2c.iask.in/mg2c_v2.0/) [69].

### 2.9. Gene Ontology Analysis of *LecRLK* Genes in Barley

We used the online tool Plant Transcription Factor Database (PlantTFDB, https://planttfdb.cbi.pku.edu.cn//) to carry out the gene ontology (GO) analysis to predict the relationship of identified *LecRLK* genes with the group of various biological processes, cellular processes, and molecular functions [70].

### 2.10. Prediction of Subcellular Localization of the Identified LecRLK Proteins in Barley

The subcellular locations of the identified LecRLK proteins were predicted in the various cell organelles by an online predictor named plant subcellular localization integrative predictor (PSI) (https://bis.zju.edu.cn/psi/) [71].

### 2.11. Regulatory Relationship between Transcription Factors and *LecRLK* Genes in Barley

To identify important transcription factors (TFs) associated with the identified *LecRLK* genes, we used the PlantTFDB 4.0 (https://planttfdb.cbi.pku.edu.cn//) [70]. Moreover, we constructed a regulatory network between *LecRLK* genes predicted TFs and visualized them by Cytoscape 3.9.1 [72].

### 2.12. Analysis of *cis*-Acting Regulatory Elements (CAREs) of *HvLecRLK* Gene Promoters

The cis-acting regulatory elements (CAREs) associated with various stress responses were predicted in the 1.5 kb upstream regions of the identified *LecRLK* genes by using a portal prediction tool with the Signal Scan search program in the PlantCARE database (https://bioinformatics.psb.ugent.be/webtools/plantcare/html/) [73]. Furthermore, predicted CAREs were divided into four classes based on their functional regulatory roles: light-responsive (LR), tissue-specific (TS), hormone-responsive (HR), and stress-responsive (SR).

### 2.13. Putative microRNA Target Site Analysis

To predict potential miRNAs targeting barley *HvlecRLK* genes, we used the default parameters of psRNATarget (https://plantgrn.noble.org/psRNATarget/analysis?function=3) by submitting CDS sequences for sequence complementary to miRNAs [74].

### 2.14. Protein-Protein Interaction Network Prediction of HvlecRLKs

We predicted the protein-protein interaction (PPI) network of HvlecRLKs using STRING version-11.0 (https://string-db.org/cgi/) database based on homologous protein from *Arabidopsis*. For PPI network analysis, STRING tool parameters were used as follows: (i) full STRING network was used as network type, (ii) the meaning of network edge evidence, (iii) interaction score was set to 0.4 (medium confidence parameter), and (iv) maximum number of interaction display is <10.

## 3. Results and Discussion

### 3.1. Identification of Lectin Receptor-Like Kinase (LecRLK) Proteins in Barley Genome

A total of 113 lectin receptor-like kinase (LecRLK) proteins in barley (*H. vulgare*) were identified using G-type, C-type, and L-type AtlecRLK protein as query sequences to build a Hidden Markov Model (HMM). Based on their domain organization, HvlecRLKs proteins were then classified as G-type HvlecRLKs, C-type HvlecRLKs, and L-type HvlecRLKs consisting of 62, 1, and 50 HvlecRLK proteins in the barley (*H. vulgare*) genome, respectively. The identified *HvlecRLK* genes, their chromosomal location, orientation, structural characteristics (ORF and gene length), and protein properties (molecular weight, protein length, and pI value) are shown in Table 1.

In G-type HvlecRLKs, ORF length ranged from 927 bp (HvleckRLK38) to 2736 bp (HvleckRLK34), encoding potential amino acid length of 309 aa and 912 aa, respectively. The genomic length of G-type HvLecRLKs varied from 2559 bp (HvleckRLK12) to 225550 bp (HvleckRLK16) and the molecular weight ranged from 32.4 kDa (HvleckRLK38) to 100.16 kDa (HvleckRLK34). Notably, G-type HvlecRLKs exhibited both acidic and basic properties based on their pI values. The highest pI value was observed for HvleckRLK56 (8.8; indicating basic properties), whereas the lowest pI value was observed for HvleckRLK38 (5.31; indicating acidic properties).

C-type HvlecRLKs (HvleckRLK63) displayed an ORF length of 1845 bp encoding a potential amino acid length of 615 aa. The genomic length and the molecular weight of the corresponding protein were 4182 bp and 67.7 kDa, respectively. C-type HvlecRLK was characterized by higher basic properties with a pI value of 9.34. Among L-type HvlecRLKs, the ORF length ranged from 1215 bp (HvleckRLK67) to 2607 bp (HvleckRLK81), encoding proteins with lengths 405 aa and 869 aa. The genomic length of L-type *HvlecRLK* genes varied between 1743 bp (HvleckRLK82) and 500635 bp (HvleckRLK73). The molecular weight ranged from 41.26 kDa (HvleckRLK67) to 95.08 kDa (HvleckRLK81). The pI value of L-type HvLecRLK varied from 5.4 (HvleckRLK86 and HvleckRLK88) to 9.14 (HvleckRLK70).

LecRLK family proteins are prevalent in plant species with their number ranging from 21 to 325. However, no clear correlation exists between the gene number and the genome size of these plant species [75]. In the case of barley, the total number of LecRLKs (113) was higher than *Arabidopsis* (*A. thaliana*) (75), shrub (*Amborella trichopoda*) (56), and corn (*Zea mays*) (95) [39]. Notably, a higher number of G-type LecRLKs were identified than L-type LecRLKs in barley (G-type: 62 vs L-type: 50), whereas in *Arabidopsis* (*A. thaliana*), L-type LecRLKs predominate over G-type LecRLks(G-type: 32 vs L-type: 42) [27]. Similar findings were also observed in *Populous* (*P. trichocarpa*) (G-type: 180 vs L-type: 50) [39] and rice (*O. sativa*) (G-type: 100 vs L-type: 72) [27].

### 3.2. Phylogenetic Relationship of LecRLK Proteins in Barley and *Arabidopsis*

**Figure 1:**
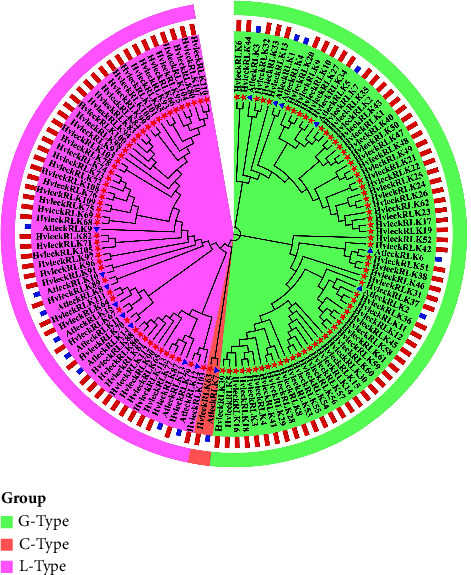
The phylogenetic relationship between barley and *Arabidopsis* LecRLK family proteins. Phylogenetic tree representing the evolutionary relationship for the G-type LecRLK, C-type LecRLK, and L-type LecRLK proteins from *H. vulgare* and *Arabidopsis*. The phylogenetic trees were constructed using the neighbor-joining method. Different groups present here are indicated by different colors. The red dots represent the *Arabidopsis* lecRLK proteins and the blue lines represent the barley lecRLK proteins.

The phylogenetic tree analysis revealed the evolutionary relationship between G-type, C-type, and L-type LecRLK proteins in barley and *Arabidopsis* with AtlecRLK protein sequences as query sequences (Figure 1). Among G-type LecRLKs, six G-type AtlecRLKs were used as the representative genes and 62 G-type HvlecRLKs were subjected to tree construction. Based on the higher sequence similarity, HvleckRLK36, HvleckRLK32, HvleckRLK33, HvleckRLK20, HvleckRLK7, HvleckRLK35, and HvleckRLK51 were clustered with AtleckRLK1, AtleckRLK2, AtleckRLK3, AtleckRLK4, AtleckRLK5, and AtleckRLK6, respectively. We also found that HvleckRLK63 (C-type HvlecRLK) formed a cluster with AtleckRLK7 (C-type AtlecRLK).

In our analysis, among 50 L-type HvlecRLK proteins, HvleckRLK89, HvleckRLK68, HvleckRLK69, HvleckRLK91, HvleckRLK67, HvleckRLK79, HvleckRLK87, HvleckRLK70, HvleckRLK64, and HvleckRLK111 formed clusters with AtleckRLK8, AtleckRLK9, AtleckRLK10, AtleckRLK11, AtleckRLK12, AtleckRLK13, AtleckRLK14, and AtleckRLK15, respectively. Notably, AtleckRLK13, AtleckRLK14, AtleckRLK11, AtleckRLK15, AtleckRLK8, AtleckRLK9, and AtleckRLK10 were found to enhance H_2_O_2_ (hydrogen peroxide) and cell death in response to a pathogenic bacteria like *Pseudomonas syringae* and pathogenic oomycetes *Phytophthora infestans* and *Phytophthora capsici* [76]. Correspondingly, the HvlecRLK proteins exhibit a high activation level in response to pathogenic resistance. Additionally, AtLecRK-VI.2 (AT5G01540) was found to induce resistance against *Pectobacterium carotovorum* and *Pseudomonas syringae* [77, 78] while AtLecRK-IV.3 (AT4G02410) was found to induce resistance against *Botrytis cinerea* [79]. Several AtLecRKs such as AtLecRK-VI.2 (AT5G01540) and AtLecRK-V.5 (AT3G59700) were indeed identified to be involved in hormone signaling (ABA) as well as stomatal immunity [77]. The majority of sequences from *A. thaliana* and *H. vulgare* are different, with only a total of 19 HvLecRLKs clustered with 15 AtlecRLKs revealing the distinct evolutionary functions of HvLecRLKs. A similar trend was previously identified in *Taxodium “Zhongshanshan”* and other herbaceous as well as many woody plants [15, 39]. Moreover, LecRLKs in various woody plants formed separate clades from each other. Thus, it might be concluded that there are significant differences between the LecRLK sequences among various species.

### 3.3. Conserved Domain Analysis of LecRLK Proteins in Barley

**Figure 2:**
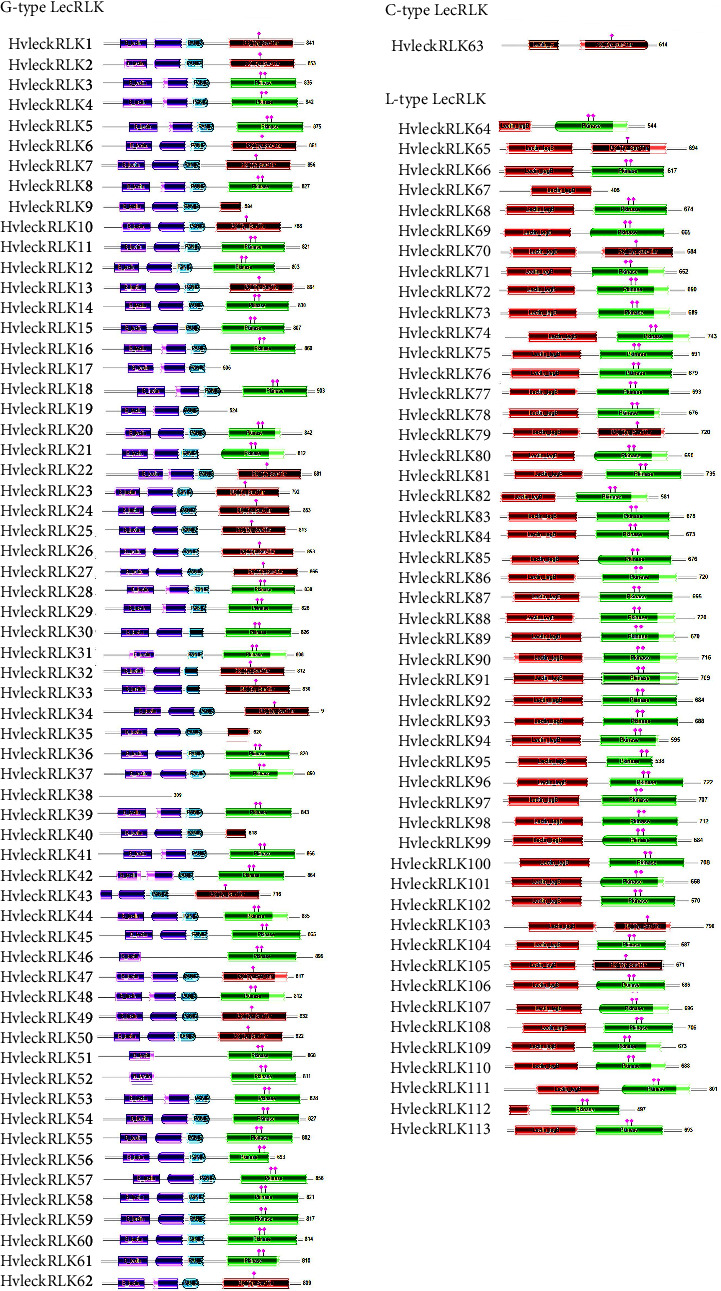
Feature domain of *Hordeum vulgare* L. LecRLK proteins. The conserved domains of the identified HvlecRLK proteins were drawn by using the Pfam database [64]. The position of the identified domain is demonstrated by different colored boxes including the domain name.

Domain organization and architecture of all HvlecRLKs were analyzed by using the conserved domain searching database HMMER, which led to the identification of three N-terminal domains: Lectin_legB (PF00139), Lectin_C (PF00059), and B_lectin (PF01453), associated with L-type, C-type, and G-type LecRLKs of barley (*H. vulgare*) (Figure 2). L-type HvlecRLKs typically contained legume lectin domain (Lectin_legB; PF00139) either with protein kinase domain (Pkinase; PF00069) or protein tyrosine and serine/threonine kinase domain (PK_Tyr_Ser-Thr; PF07714). Only one member of L-type HvlecRLKs (HvleckRLK67) was noticed to contain the Lectin_legB (PF00139) domain alone while 44 out of 50 L-type HvlecRLKs contained Pkinase conserved domain (PF00069) with the remaining 5 members possessing the PK_Tyr_Ser-Thr domain (PF07714) in addition to Lectin_legB domain (PF00139). Both the Lectin_legB domain (PF00139) and kinase domain (PF00069) were also detected in L-type LecRLKs of *Taxodium “Zhongshanshan”* [15] Due to the resemblance of the L-type LecRLK domain to legume lectins, it is anticipated that L-type HvlecRLKs may be involved in signal identification and transduction [38]. Barley (*H. vulgare*) contained a single member of C-type LecRLKs which carried the lectin C-type domain (Lectin_C; PF00059) as well as the PK_Tyr_Ser-Thr conserved domain (PF07714). However, two C-type LecRLKs were observed in *Taxodium “Zhongshanshan”* containing lectin-C domain (PF00059) and kinase domain (PF00069) [15].

Domain architecture of G-type HvlecRLKs was more complex compared to C-type and L-type HvlecRLKs. G-type HvlecRLKs were found to have usually D-mannose binding lectin domain (B_lectin; PF01453), S-locus glycoprotein domain (S_locus_glycop; PF00954), Protein tyrosine and serine/threonine kinase domain (PK_Tyr_Ser-Thr; PF07714), PAN-like domain (PAN_2; Pfam accession number was not detected) [41], and protein kinase domain (Pkinase; PF00069). A total of 23 G-type HvlecRLKs exhibited four domains including PK_Tyr_Ser-Thr (PF07714) along with B-lectin (PF01453), S_locus_glycop (PF00954), and PAN2. In an alternative manner, 33 G-type HvlecRLKs contained Pkinase (PF00069) with B-lectin (PF01453), S_locus_glycop (PF00954), and PAN_2 domain. However, two G-type HvlecRLKs (HvleckRLK17 and HvleckRLK19) carried three domains: B_lectin (PF01453), S_locus_glycop (PF00954), and PAN_2 domains, while three G-type HvlecRLKs (HvleckRLK46, HvleckRLK51, and HvleckRLK52) contained only B_lectin domain (PF01453) and Pkinase domain (PF00069). Remarkably, 57 out of 62 G-type HvlecRLKs featured the S_locus_glycop domain (PF00954) which is known for its significant role in self-incompatibility response [80]. The presence of the PAN-2 domain in most G-type HvlecRLKs (58 out of 62) suggests their involvement in protein-protein and/or protein-carbohydrate interaction [28, 81, 82]. Several N-terminal domains such as S_locus_glycop (PF00954), EGF (PF12947), and PAN_2 were also identified in StLecRLKs of potato (*Solanum tuberosum* L.). Additionally, DUF3660 (PF12398) and DUF3403 (PF11883), two intracellular domains, were observed in StLecRLKs [41]. In cucumber (*C. sativus* L.), among 24 G-type CsLecRLKs, both PAN and EGF domains (PF12947) were detected in 10 CsLecRLKs, only PAN domain (PF00024) was observed in 5 proteins, and only EGF domains (PF12947) were found in 8 proteins. However, one protein was detected to lack both the PAN domain (PF00024) and the EGF domain (PF12947) showing similarity to our identified G-type HvlecRLK38 containing no PAN or EGF domain (PF12947) [42]. Our findings also align with the previous investigation on LecRLKs of *Taxodium “Zhongshanshan”* containing all four basic domains: B-lectin domain (PF01453), kinase domain (PF00069), S-locus glycoprotein (PF00954), and PAN domain (PF00024) [15]. A higher number of G-type HvlecRLKs imply their diverse role in plant development and response to environmental stimuli.

### 3.4. Conserved Motif Analysis of LecRLK Proteins in Barley

**Figure 3:**
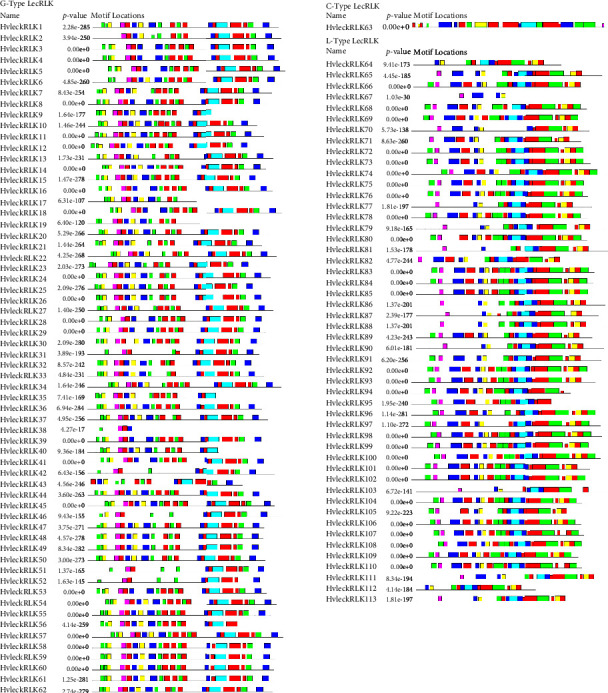
The distribution of conserved motifs in barley LecRLK protein. The distribution of conserved motifs of the predicted G-type, C-type, and L-type HvlecRLK protein families is illustrated using MEME-suite (https://meme-suite.org/meme/) (a maximum of 20 motifs are displayed) [65]. Each color represents different motifs within the predicted protein domains.

The motifs are very short active sites of enzymes facilitating the mechanism of protein folding [83]. To explore conserved motifs in HvlecRLKs, the MEME program was used and identified 20 conserved motifs distributed among G-type, C-type, and L-type LecRLKs in barley, ranging from 04 to 20 motifs (Figure 3). In G-type HvlecRLK, 15 of them displayed the maximum number of motifs (20 motifs) indicating higher similarity with AT4G21380 (20 motifs) and were assumed to perform alike. However, the lowest number of motifs was identified in HvleckRLK38 (04 motifs). C-type LecRLK HvleckRLK63 featured 20 motifs that were similar to the paralog AtleckRLK7. In L-type HvLecRLKs, 20 conserved motifs were predicted in 14 HvLecRLKs each while HvleckRLK67 contained only 4 conserved motifs. L-type AtleckRLK10 and AtleckRLK9 had 18 motifs that exhibited higher conservation with HvleckRLK66, HvleckRLK68, and HvleckRLK96 each having 18 conserved motifs. This variation in motif numbers may contribute to the functional assortment between barley (*H. vulgare*) and *Arabidopsis* (*A. thaliana*). Similar motif patterns have been found in CslecRLKs of cucumber (*C. sativus*) and *Cerasus humilis* showing distinct motif features related to the variations in their protein sequences. In total, 10 conserved motifs were observed in CslecRLKs ranging from 4 to 10 in each protein and 14 conserved motifs in *Cerasus humilis* [84, 85]. Motifs 1 to 5 were predominantly identified in L-type CsLecRLK, whereas motif 1, motif 2, motif 6, and motif 8 were frequently observed in G-type CsLecRLK protein [84]. The variations in motif organizations indicated the functional diversity of the associated proteins.

### 3.5. Gene Structure Analysis of *LecRLK* Genes in Barley

**Figure 4:**
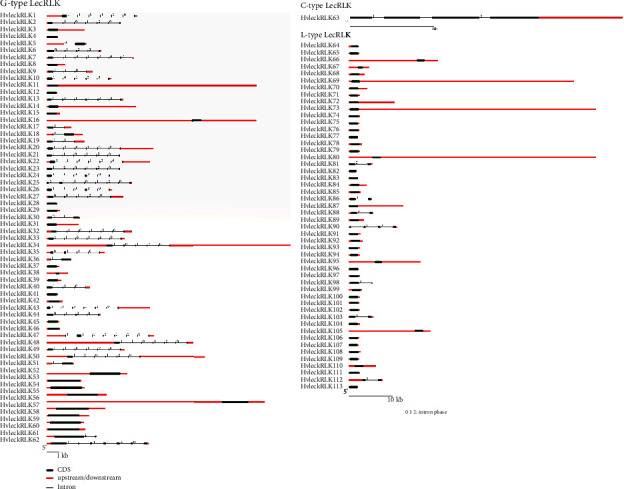
The gene structure of barley *LecRLK* genes. Gene structure of the predicted G-type, C-type, and L-type *LecRLK* genes in *H. vulgare* compared to *A*. *thaliana* is illustrated using Gene Structure Display Server (GSDS 2.0, https://gsds.cbi.pku.edu.cn/index.php) [66]. Gene families are categorized based on their phylogenetic relationship. For all *HvlecRLK* genes, black lines represent introns, green-bold lines represent exons, and red-bold lines represent 5′ and 3′ untranslated regions (UTR). The gene structure of each *HvlecRLK* is displayed according to the scale mentioned at the bottom.

Evaluation of *HvlecRLK* gene structures revealed the exon-intron configuration of the G-type, C-type, and L-type *HvlecRLK* genes which displayed higher conservation compared to the corresponding reference *AtlecRLK* genes (Figure 4). In this study, we observed that 61.95% of *HvlecRLKs* (70 out of 113) were intron-less. The highest number of introns (7 introns) was identified in *HvleckRLK7*, *HvleckRLK25*, and *HvleckRLK47* belonging to the G-type LecRLK subfamily. Among the 62 G-type *HvlecRLKs*, 27 genes had no intron while the remaining exhibited a variable number of introns. Some members of *HvlecRLK* exhibited similar exon-intron organization while many had a lower number of introns compared to G-type *AtlecRLK*. C-type *HvlecRLK* carrying 4 exons and 3 introns was just one less than C-type *AtlecRLK*. Most L-type *HvlecRLKs* exhibited structural similarity to the corresponding *Arabidopsis* (*A. thaliana*) genes. Notably, 43 members had no intron while 6 members (*HvleckRLK81*, *HvleckRLK86*, *HvleckRLK88*, *HvleckRLK98*, *HvleckRLK103*, and *HvleckRLK112*) carried only one intron. The maximum intron number of L-type *HvlecRLK* (3 introns) was found in *HvleckRLK90*. The well-conserved gene structure of *HvlecRLK* genes with *Arabidopsis* (*A. thaliana*) suggests similar functional activity.

The gene structure analyses revealed that the average number of intron per *HvlecRLKs* was 1.5, significantly lower than that in cucumber genes (4.39 introns per gene) [86]. A similar phenomenon has been observed in other plants. For instance, most *LecRLK* genes in soybeans (*G. max*) contained either one intron or none at all [34]. Previous investigations also identified introns in only a few *LecRLK* genes in *Arabidopsis* (*A. thaliana*) and rice (*O. sativa*). For example, out of the 75 *LecRLK* genes in *Arabidopsis* (*A. thaliana*) and 173 *LecRLK* genes in rice (*O. sativa*), only five and eight genes contained intron, respectively [27]. Gene structure analysis revealed the divergence of G-type, C-type, and L-type *HvlecRLK* genes. For instance, there are mainly 8 gene structure groups according to the number of introns (0 to 7 introns). However, in *GmlecRLKs* of *G. max*, four gene structure groups were identified containing 3 introns, six introns, seven introns, and no introns in their coding sequences [34]. It has been previously demonstrated that introns play a pivotal role in cellular processes as well as plant developmental processes by regulating gene expression or alternative splicing [87]. Notably, most of the L-type *LecRLKs* in both *H. vulgare* and *G. max* have no intron demonstrating that they are more conserved and showed less divergence in structure [34]. The compact gene structure is expected to enhance transcriptomic gene expression by inhibiting variable splicing and reducing energy consumption, particularly for genes responding to various environmental stresses.

### 3.6. Ka/Ks Analysis of HvlecRLK Gene Family

**Figure 5:**
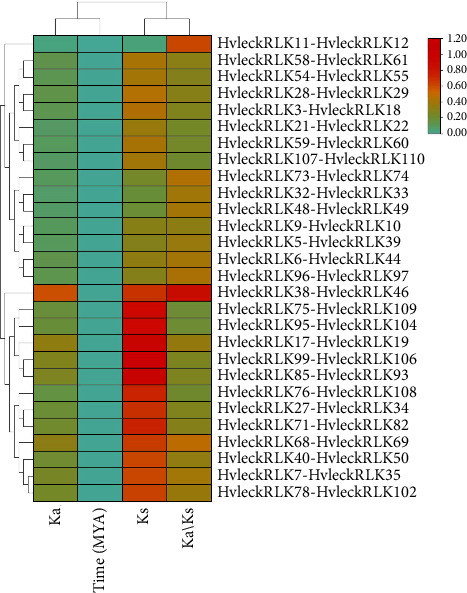
The Ka/Ks analysis of *HvlecRLK* genes. The gene duplication period of *HvlecRLK* duplicated gene pairs was estimated using Ka and Ks values. Ka values represent the number of nonsynonymous substitutions per nonsynonymous site, while Ks values represent the number of synonymous substitutions per site. The ratio of Ka to Ks changes is represented by Ka/Ks.

The values of Ka (nonsynonymous substitutions) and Ks (synonymous substitutions) and Ka/Ks ratios were analyzed to determine the selection pressure and evolutionary history of *lecRLKs* in barley (*H. vulgare*) (Figure 5). In total, 28 homologous pairs of *HvlecRLKs* were determined. During the evolutionary period, genes evolved from various selection pressures, such as purifying selection, natural selection, and positive selection. Our investigation determined the Ka/Ks ratios for 28 *HvlecRLK* duplicated pairs ranging from 0.19 (*HvleckRLK75-HvleckRLK109*) to 0.86 (*HvleckRLK38-HvleckRLK46*) indicating the evolution through purifying selection of these paired genes. The Ka/Ks ratios of all duplicated *lecRLK* genes in soybean (*G. max*) were less than 0.5, also suggesting evolution through purifying selection [34]. However, in cucumber (*C. sativus*) [84] and peanut (*Arachis hypogaea*) [88], both positive and purifying selections were determined in duplicated *CslecRLK* and *AhlecRLK* genes. Furthermore, we analyzed the divergence period of duplicated *HvlecRLKs* ranging from 1.25E-16 (*HvleckRLK11*-*HvleckRLK12*) to 1.09E-15 (*HvleckRLK6-HvleckRLK44*) with an average duplication time of 1.74E-15 MYA, demonstrating the recent gene duplication events of *HvlecRLK*s in barley (*H. vulgare*). Similar findings were also observed in *AhRLK* genes of *Arachis hypogaea* in which the divergence period ranged from 0 to 2 MYA illustrating their evolution through recent gene duplication events [88]. It might be concluded that *HvlecRLK*s underwent duplication before their existence with several potential functions.

### 3.7. Collinearity and Synteny Analysis of the LecRLK Gene Family in Barley

**Figure 6:**
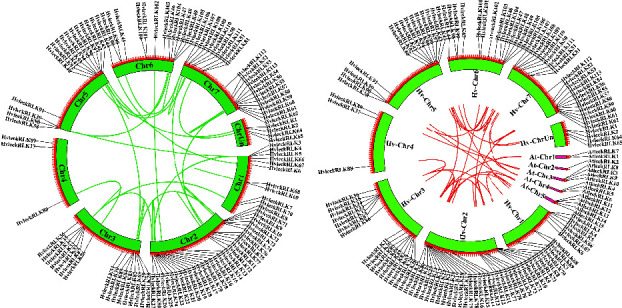
The collinearity and syntenic relationships between barley (*H. vulgare*) and *Arabidopsis* (*A. thaliana*). (a) The collinearity analysis of the LecRLK gene family in barley. The colored rectangles represent chromosomes 1–7 with an unknown chromosome. The collinear blocks are represented with colored lines. (b) The synteny analysis of *LecRLK* genes between barley and *Arabidopsis*. The colored rectangles represent chromosomes 1–7 with an unknown chromosome and the red colored lines represent the synteny blocks.

To determine the evolutionary relationship between the lecRLK gene family of barley and *Arabidopsis*, a comprehensive collinearity analysis was conducted (Figure 6(a)). Collinearity, a particular form of synteny, requires specific gene order [89]. This investigation showed that 34 collinear pairs were identified within *HvlecRLK* genes, with the highest number of collinear genes found in chromosome 2 (12) followed by chromosome 7 (09), chromosome 3 (08), chromosome 5 (07), chromosome 6 (06), and chromosome 1 (05). Furthermore, two collinear genes were identified in an unknown chromosome and the least number was observed in chromosome 4 (01). These collinear *HvlecRLK* gene pairs were involved in lineage-specific expansion over evolution [90]. Moreover, synteny analysis was also conducted to reveal the expansion mechanism and evolutionary relationship of the lecRLK gene family between barley and *Arabidopsis* genome (Figure 6(b)). In total, 7 syntenic gene pairs were identified showing higher homology with *AtlecRLKs*. The syntenic analysis was also previously performed in cucumber *lecRLK* genes identifying higher homology between *CslecRLKs* and *AtlecRLK* [84]. This study suggests that the *HvlecRLK* genes were highly conserved having similar ancestors with which performed similar functions.

### 3.8. Analysis of Chromosomal Location of *LecRLK* Genes in Barley

**Figure 7:**
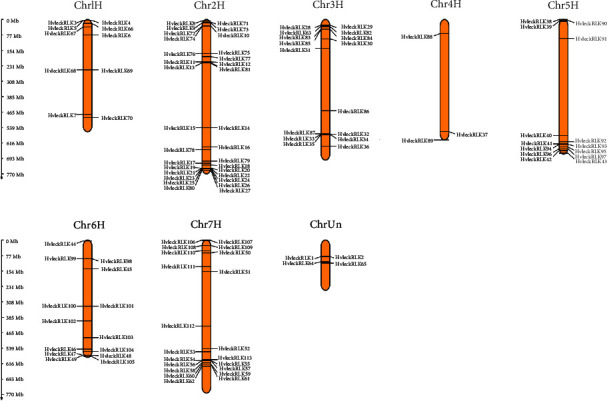
The chromosomal location of *HvlecRLK* genes. The chromosomal location of the predicted *HvlecRLK* genes is illustrated. The chromosome number is at the top of each chromosome bar. The scale to indicate the chromosomal length as millions of bases (Mb) is provided on the left based on the information retrieved from Phytozome v13 [54]. ChrUn means the unknown chromosome.

We investigated the chromosomal locations of barley *LecRLKs* to understand the genomic distribution of the predicted genes (Figure 7). This study revealed that mapped G-type, C-type, and L-type *HvlecRLK* genes were located on 8 individual chromosomes including an unknown chromosome (ChrUn) within 770 Mb in the entire genome of barley (*H. vulgare*) (Figure 5). The number of *HvlecRLKs* on each chromosome ranged from 3 to 31, with Chr2H containing the highest number of *HvlecRLKs* (31) while chr4H had only 3 *HvlecRLKs*. Four *HvlecRLKs* were identified in an unknown chromosome. All 62 G-type *HvlecRLK* genes were distributed across 8 independent chromosomes, with 5, 20, 9, 01, 6, 6, and 13 *HvlecRLKs* in Chr1H to Chr7H, respectively. Two G-type *HvlecRLKs* (*HvleckRLK1*, *HvleckRLK2*) were found on ChrUn. A single C-type *HvlecRLK* gene was located on Chr3H (*HvleckRLK63*). Among the 50 L-type HvlecRLKs, number 5, 11, 6, 2, 8, 8, and 8 HvlecRLKs were unevenly distributed on Chr1H-Chr7H, respectively, while HvleckRLK64 and HvleckRLK65 were located on an unknown chromosone (designated as ChrUn). Our finding showed similarity to previous investigations on *LecRLKs* of cucumber (*C. sativus*) [42], potato (*S. tuberosum*) [41], and soybean (*G. max*) [34] in which *LecRLK* genes were unevenly scattered on a total of 7, 12, and 19 chromosomes, respectively. In cucumber, the highest number of *CslecRLKs* (12) was located on chromosome 3 while in potato, the largest number of *StlecRLk*s (20) was identified on chromosome 7 [41, 42]. However, In *G. max*, chromosome 4 and chromosome 18 contained only G-type and L-type *GmlecRLKs*, separately, and 17 chromosomes consisted of both G-type and L-type *GmlecRLKs*. Additionally, the largest number of *GmlecRLks* was located on chromosome 6, chromosome 12, and chromosome 13 [34]. Furthermore, C*hLecRLK* genes of *C. humilis* were found to be unevenly distributed through eight chromosomes consisting of the majority of *ChLecRLK* genes (56) on chromosome 3 and lowest on chromosome 8 (3) [85].

### 3.9. Gene Ontology Analysis of *LecRLK* Genes in Barley

**Figure 8:**
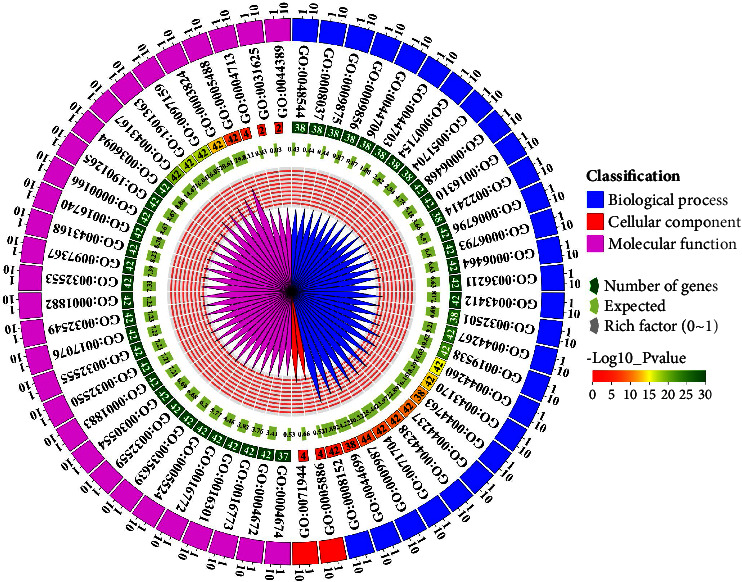
The gene ontology (GO) terms correspond to *HvLecRLK* genes. The predicted GO terms corresponding to the reported *HvlecRLK* genes are presented for biological processes, cellular components, and molecular functions whether the genes are associated or not. The *p* value corresponding to the GO terms is shown in the histogram, using -log10 (*p* value).

To gain insight into the various cellular, molecular, and biological functions of *LecRLK* genes, we conducted a gene ontology (GO) analysis (Figure 8). Since most *HvlecRLKs* were associated with three categories of GO terms including biological process, molecular functions, and cellular components, the total number of *HvlecRLKs* and GO terms may not match each other. In biological processes, the highest number of GO annotation was involved in “metabolic process” (GO:0008152; *p* value: 6.40E-10) and also showed higher representation in phosphorus metabolic process (GO:0006793; *p* value: 1.00E-30), protein metabolic process (GO:0019538; *p* value: 1.00E-30), cellular metabolic process (GO:0044237; *p* value: 1.70E-21), phosphate-containing compound metabolic process (GO:0006796; *p* value: 1.00E-30), and organic substance metabolic process (GO:0071704; *p* value: 9.20E-18). In this category, *HvlecRLKs* were also associated with the primary metabolic process (GO:0044238; *p* value: 7.40E-20) including the macromolecule metabolic process (GO:0043170; *p* value: 1.80E-29). Additionally, *HvlecRLks* were also associated “protein modification process” (GO:0036211; *p* value: 1.00E-30) and “protein phosphorylation” (GO:0006468; *p* value: 1.00E-30). Our study is supported by a previous investigation on potatoes (*S. tuberosum*) which found that a larger number of LecRLK family members were implicated with the “metabolism process” and “protein modification process” [41].

Additionally, *HvlecRLKs* were also implicated in “pollination” (GO:0009856; *p* value: 1.00E-30), “recognition of pollen” (GO:0048544; *p* value: 1.00E-30), and “pollen-pistil interaction” (GO:0009875; *p* value: 1.00E-30) suggesting the involvement of these genes in pollination process. Some studies have indicated the importance of *LecRLK* in the self-incompatibility of flowering and pollination [91, 92]. Interestingly, 2 different genes (*HvleckRLK111* and *HvleckRLK113*) were identified to take part in the “defense response to oomycetes” (GO:0002229; *p* value: 0.0062) and “response to oomycetes” (GO:0002239; *p* value: 0.0071). Existing studies also support the role of *LecRLK* genes in interaction with oomycetes [23, 93, 94] and fungi [79]. Among molecular functions' GO terms, *HvlecRLK* genes were strongly associated with “kinase activity” (GO:0016301; *p* value: 1.00E-30), “ATP binding” (GO:0005524; *p* value: 1.00E-30), “ion binding” (GO:0043167; *p* value: 1.00E-30), “catalytic activity” (GO:0003824; *p* value: 1.70E-26), and “transferase activity” (GO:0016740; *p value*: 1.00E-30). However, the lowest number of GO annotations was associated with the “cellular process” GO term and “cell periphery” (GO:0071944; *p* value: 0.00012) and “plasma membrane” (GO:0005886; *p* value: 3.80E-05) GO terms. This is consistent with previous investigation, which reveals that lectins are not only found on the plasma membrane but also in the nucleus and cytoplasm [95]. Thus, our GO analysis indicates the extensive functions, processes, and cellular localizations of *HvlecRLK* genes and may pave the way to identifying additional functions of the lectin gene family.

### 3.10. Prediction of Subcellular Localization of the Identified LecRLK Proteins in Barley

**Figure 9:**
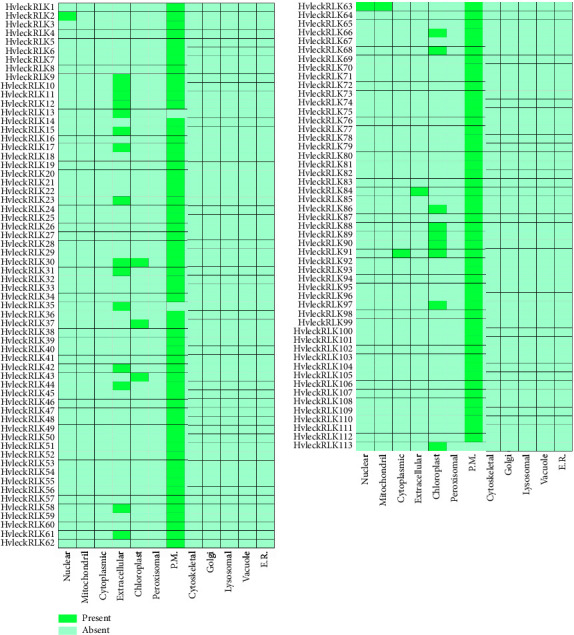
A heatmap represents the subcellular localization of barley HvlecRLK protein. Subcellular localizations for the G-type, C-type, and L-type HvlecRLK proteins are shown in the heatmap. The names of each HvlecRLK protein are displayed on the left side of the heatmap, with the terms of the respective cellular organelles displayed at the bottom. The color intensity on the right side of the heatmap shows the presence of protein signals associated with the genes. In this study, reported proteins were analyzed in the plasma membrane, extracellular region, chloroplast, nucleus, mitochondria, and cytoplasmic region.

The study of subcellular localization revealed the cellular appearance of the reported proteins. In this investigation, the majority of HvlecRLK proteins were predicted in the plasma membrane (G-type HvlecRLK is 96.77%, C-type HvlecRLK is 100%, and L-type HvlecRLK is 98%) followed by extracellular region (G-type HvlecRLK is 24.19%, C-type HvlecRLK is 0%, and L-type HvlecRLK is 2%) and chloroplast (G-type HvlecRLK is 4.83%, C-type HvlecRLK is 0%, and L-type HvlecRLK is 18%) (Figure 9). The LecRLK proteins located in the plasma membrane play roles in connecting the cell wall and membrane, facilitating transmembrane movements, and ultimately regulating plant responses to pathogen attacks [84]. However, we observed that one G-type HvlecRLK, HvleckRLK2, appeared in the nuclear region and one L-type HvlecRLK, HvleckRLK91, appeared in the cytoplasmic region. It is worth noting that C-type HvlecRLK was also found in the nucleus and mitochondria. Previous studies have shown that LecRLK proteins present in mitochondria play a crucial role in plant growth and stress response mechanisms [96]. The majority of ThzlecRLKs proteins (71.7%) in Taxodium “Zhongshanshan” and StlecRLKs proteins (77%) in S. lycopersicum were located in the plasma membrane which also support our finding subcellular localization analysis [15, 41]. The remaining LecRLKs are present in other cellular loci such as mitochondria, chloroplast, vacuole, and nucleus. According to the result, we can speculate that the HvlecRLks are not limited to the cell membrane but the other cellular organelles. Thus, the HvlecRLKs found in several loci might be expressed in the whole cell system.

### 3.11. Regulatory Relationship between Transcription Factors and *LecRLK* Genes in Barley

**Figure 10:**
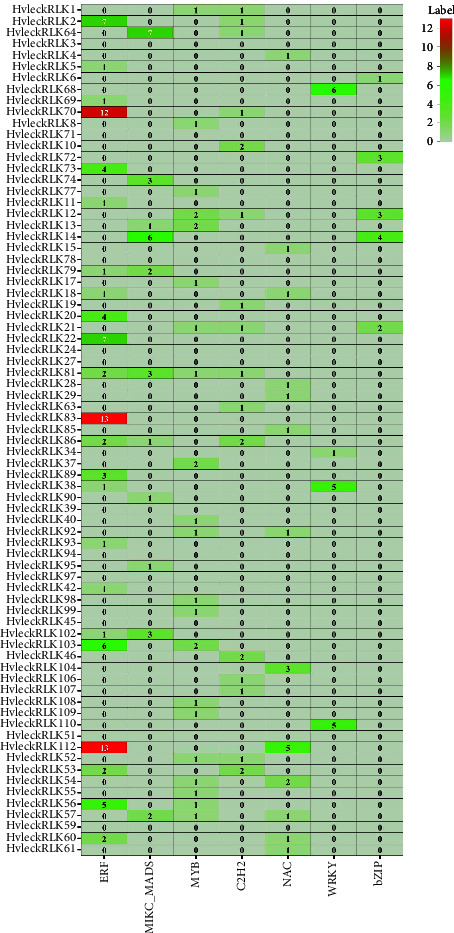
The distribution of transcription factors on the promoter region of *HvLecRLK* genes. *LecRLK *gene-mediated subnetwork for bZIP, C2H2, ERF, MIKC_MADS, MYB, NAC, and WRKY TFs families which is expressed as heatmap. The name of each gene is shown on the left side of the heatmap.

Transcription factors (TFs) play a pivotal role in regulating different biological processes including plant stress response, defense, metabolism, and developmental processes [97–99]. In plants, numerous TFs (AP2, Dof, NAC, MYB, MIKC_MADS, ERF, bZIP, C2H2, and WRKY) have been identified in response to various environmental stimuli and developmental stages (Figure 10) [99–103]. A total of 381 TFs were found regulating the functions of candidate *LecRLK* genes in the barley genome. These identified TFs were categorized into 29 different families. Notably, the main 7 TF families including ERF, NAC, MYB, WRKY, bZIP, MIKC_MADS, and C2H2 families accounted for 52.2% of all the identified TFs (Figure 10). These TFs demonstrated a unique structure and connected to the candidate *LecRLK* genes based on network and subnetwork analysis. The dominant TF family (TFF) ERF had a connection with 23 *HvlecRLKs* containing a total of 91 transcription factor binding sites (TFBS) and was abundant in *HvlecRLK70*, *HvlecRLK83*, and *HvlecRLK112*. Similarly, NAC, MYB, WRKY, bZIP, MIKC_MADS, and C2H2 TF families were associated with 13, 21, 4, 5, 11, and 16 *HvlecRLK* genes, respectively. However, no major TF was identified in the promoter region of 3 L-type and 10 G-type *HvlecRLK* genes. The maximum number of TFF (8 TFF) was linked to the promoter region of both L-type *HvleckRLK64* (AP2, ARF, BBR-BPC, C2H2, Dof, G2-like, HSF, and MIKC_MADS), and *HvleckRLK86* (BBR-BPC, C2H2, CPP, EIL, ERF, G2-like, HD-ZIP, and MIKC_MADS). Additionally, five TFFs interacted with L-type *HvleckRLK112*, which contained the highest number of TFBS (23 TFBS).

The ERF TFF was recognized as one of the largest families which have been previously determined [104]. ERF family members play a crucial role in plant hormonal response under stressful conditions including response to abscisic acid and ethylene to activate stress-responsive genes and enhance salt and drought tolerance response in tomato [105, 106]. The WRKY family is known for its role in boosting defense mechanisms against pathogens in various plant species [107, 108]. Both bZIP and TFF control gene expression for plant development under abiotic stress [109, 110]. The MIKC-MADS TFF includes members with diverse functions in vegetative and reproductive phases, regulating genes associated with pollen, flower, endosperms, and root development [111]. Another important TFF C2H2 having a finger-like structure can bind zinc ions and respond to environmental stimuli [112]. On the other hand, MYB TFF is involved in cell identity, seed, and flower development, defense and stress responses, and primary and secondary metabolism regulation [113–115]. In plants, Dof TFF (DNA-binding one finger) plays a pivotal role in transcriptional regulation due to its dual functionality in binding to both DNA and proteins [116, 117]. Furthermore, it contributes to seed maturation and germination, plant hormone regulation, and resistance response to various stresses [116–118]. The enrichment of TFF might be a major source of functional diversity in plant genomes [119]. The interaction between TFs and the identified genes in barley represents an extensive variability of gene expression pattern which can be explored thoroughly by further investigation in wet lab experiments.

### 3.12. Analysis of *cis*-Acting Regulatory Elements (CAREs) of *HvlecRLK* Gene Promoters

**Figure 11:**
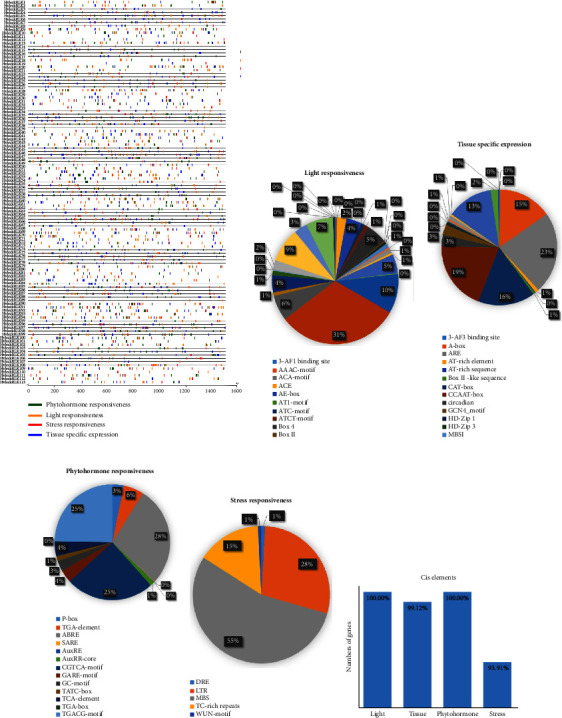
The distribution of cis-regulatory elements in the promoter region of the identified G-type, C-type, and L-type *HvLecRLK* genes. (a) The distribution of cis-regulatory elements in the *HvlecRLK* promoter region is illustrated as a heatmap. The names of each *HvlecRLK* gene are displayed on the left side of the heatmap. The green, orange, red, and blue colors represent CAREs of corresponding *HvLecRLK*s such as light responsiveness (LR), tissue-specific (TS), phytohormone responsiveness (HR), and stress responsiveness (SR), respectively. The percentage (%) ratio of the numerous cis-elements from each category is presented in pie charts: (b) light-responsive; (c) tissue-specific; (d) phytohormones-responsive; (e) stress-responsive. (f) The percentage (%) of *HvlecRLK* genes involved in four categories of cis-elements.

The cis-acting regulatory elements (CAREs) mainly consist of short DNA motifs (5–20 bp) located in the promoter region of the target gene. The CAREs predicted in the gene promoter provide valuable information about their roles in plant growth, development, and stress response [120]. Our analysis identified a total of 12648 cis-elements belonging to 75 CARE motifs including 36 different types of CARE motifs associated with light-responsive (LR) functions, 21 tissue-specific (TS) functions, 13 hormone-responsive (HR) functions, and 5 stress-responsive (SR) functions in the promoter regions of *HvlecRLKs* (Figure 11(a)). When comparing with all four motif categories, the highest number of cis-elements was detected in HR categories at 39.60%, followed by LR at 32.15%, TS 21.17%, and SR 7.09%. These cis-elements play a vital role in plant defense mechanisms and various stress responses [121–123]. On the other hand, CARE motifs belonging to the LR categories were abundant in the *HvlecRLKs* promoter region which is associated with photosynthesis. Photosynthesis is an important physiological process influenced by the light response in barley leaf tissue [124]. LR motifs such as G-box (31.31%), G-Box (10.01%), Sp1 (8.73%), GT1-motif (6.49%), and TCT-motif (6.98%) were predominantly found in 101, 99, 89, 67, and 63 *HvlecRLK* genes, respectively (Figure 11(b)). Notably, the highest number of LR motifs was found in the regulatory region of *HvleckRLK11* (25 motifs), *HvleckRLK50* (24 motifs), *HvleckRLK73* (24 motifs), and *HvleckRLK80* (24 motifs), respectively. Previous research has also demonstrated the significant role of these LR motifs in the light response of various plant species [124–127].

Additionally, among all TS categories motifs, ARE (22.82%), CCAAT-box (19.39%), CAT-box (15.91%), A-box (15.02%), and O2-site (12.96%) were abundantly present in the promoter region of *HvlecRLKs* (Figure 11(c)). Furthermore, we identified HR-related motifs such as CGTCA-motif (24.74%), TGACG-motif (24.74%), ABRE (28%), and TGA-element (5.73%) which were highly shared by 111, 111, 110, and 84 *HvlecRLK* genes, respectively (Figure 11(d)). *HvleckRLK80* (12 motifs), *HvleckRLK16* (11 motifs), and *HvleckRLK95* (12 motifs) dominantly shared most of the predicted HR motifs in their promoter region, indicating a strong hormonal response in plants. Phytohormones, known as plant growth regulators, play significant roles either individually or coordinately in plant growth and development [128–130]. Furthermore, we predicted the presence of LTR (28.54), MBS (54.63%), TC-rich repeats (15.16%), DRE (0.89%), and WUN (0.78%) in the *HvlecRLKs* promoter, which are known stress-responsive (SR) motifs in various plants (Figure 11(e)) [131–135]. Several *HvlecRLk* genes, such as *HvleckRLK14*, *HvleckRLK18*, *HvleckRLK33*, *HvleckRLK50, HvleckRLK52, HvleckRLK56*, and *HvleckRLK110*, shared four SR-related motifs indicating their potential response in environmental stresses. A large number of CAREs were also previously identified in *StLecRLKs* responsive to stress and phytohormones. Most of the *StLecRLKs* were phytohormone responsive which aligns with our findings [41]. In cucumber, most of the genes were highly involved in light regulation, followed by hormone responsiveness and other essential CAREs. Additionally, *CslecRLKs* are also responsive to stress such as heat, low temperature, and drought deducing multiverse functions against stresses [84]. Moreover, light and hormone-responsive elements were identified in all 113 *HvlecRLK* genes. However, tissue-specific elements and stress-responsive elements were detected on 99.1% and 93.91% *HvlecRLk* genes (Figure 11(f)). Thus, the CAREs shared by the predicted barley (*H. vulgare*) LecRLK family will provide significant insight into their function in plant development and defense mechanisms.

### 3.13. Putative microRNA Target Site Analysis

**Figure 12:**
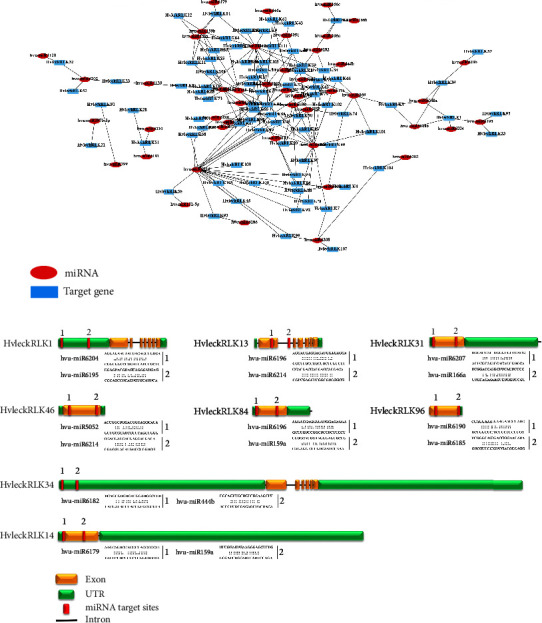
Predicted miRNAs targeted *HvlecRLK* genes. (a) Network illustration of predicted miRNA targeting *HvlecRLK* genes. Light blue rectangles represent the putative miRNAs and red oval shapes represent the targeted *HvlecRLK* genes. (b) The schematic diagram represents the *HvlecRLK* genes targeted by miRNAs and the red color represents the putative miRNAs sites of each gene.

Various studies have previously revealed the involvement of miRNAs in regulating plant signaling mechanisms, developmental processes, stress responses, and gene expressions [136–138]. Thus, to clarify the regulatory functions of miRNAs involved in *HvlecRLKs* gene regulations, 46 putative miRNAs were retrieved targeting 81 *HvlecRLKs* of 113 *HvlecRLks* genes illustrated as a network (Figures 12(a) and 12(b) and Supplementary Table 1). The retrieved miRNAs varied from 1 to 8 in numbers targeting each *HvlecRLK* gene and ranging from 20 to 24 nucleotides. Our study identified hvu-miR6204, hvu-miR6214, hvu-miR6196, and hvu-miR169 as highly abundant miRNAs and hvu-miR6204 targeted the 19 *HvlecRLks* (*HvlecRLks13, HvlecRLks36, HvlecRLks45, HvlecRLk46, HvlecRLk58, HvlecRLk68, HvlecRLk78, HvlecRLk86, HvlecRLks88, HvlecRLk89, HvlecRLK91, HvlecRLk92, HvlecRLk93, HvlecRLk94, HvlecRLk96, HvlecRLk99, HvlecRLk100, HvlecRLk105,* and *HvlecRLk109*) (Table 2). Furthermore, the hvu-miR6214 targeted 17 *HvlecRLKs* (*HvlecRLK2, HvlecRLK7, HvlecRLK15, HvlecRLK34, HvlecRLK37, HvlecRLK42, HvlecRLK44, HvlecRLK66, HvlecRLK69, HvlecRLK78, HvlecRLK87, HvlecRLKs90, HvlecRLK92, HvlecRLK96, HvlecRLK97, HvlecRLK101,* and *HvlecRLK102*) followed by hvu-miR6196 and hvu-miR169 which targeted 16 *HvlecRLKs* (*HvlecRLK6, HvlecRLK9*, *HvlecRLK13*, *HvlecRLK14*, *HvlecRLK34*, *HvlecRLK46*, *HvlecRLK63*, *HvlecRLK71*, *HvlecRLK72*, *HvlecRLK82*, *HvlecRLK83*, *HvlecRLK84*, *HvlecRLK89, HvlecRLK103*, *HvlecRLK106*, and *HvlecRLK111*) and 12 *HvlecRLKs* (*HvlecRLK6,. HvlecRLK8, HvlecRLK10*, *HvlecRLK11*, *HvlecRLK27*, *HvlecRLK55*, *HvlecRLK63*, *HvlecRLK66*, *HvlecRLK73*, *HvlecRLK87*, *HvlecRLK92,* and *HvlecRLK108*), respectively. Among all targeted genes, *HvleckRLK13* was targeted by 8 miRNAs including hvu-miR6196, hvu-miR6198, hvu-miR6214, hvu-miR168-5p, hvu-miR5053, hvu-miR6181, hvu-miR6187, and hvu-miR6189, whereas *HvleckRLK96* was targeted by 7 putative miRNAs (hvu-miR6190, hvu-miR168-5p, hvu-miR5053, hvu-miR6184, hvu-miR6185, hvu-miR6207, and hvu-miR6214).

Recently, numerous miRNAs have been retrieved from various plant species, including soybean (*G. max*) [144], *Arabidopsis* (*A. thaliana*) [145] maize (*Zea mays*) [146], rice (*O. sativa*) [147], cowpea (*Vigna unguiculata*) [148], peanut (*Arachis hypogaea*) [149], and apple (*Malus pumila*) [150], involved in plant growth, development, metabolism, and stress responses. Our results identified miR6204 as the most abundant miRNA targeting higher number of genes. miR6204 might target the genes of the SAUR-like auxin-responsive protein family, responsible for auxin metabolism [139]. The hvu-miR6214 miRNA was found abundantly and previously implicated in inducing stress response as well as antioxidant system [140]. Another abundant miRNA hvu-miR6196 has been reported to play a pivotal role in salt stress treatment [141]. Furthermore, hvu-miR169 miRNA is differentially expressed under potassium (K) stress regulating various photosynthetic processes [142]. Another research identified that miR169 in soybean, wheat, and maize was involved in plant stress tolerance in various nitrogen (N) levels [143]. This investigation suggested that the retrieved *HvlecRLKs* respond to various stress conditions by modulating the transcriptional levels of *LecRLK* genes in barley (*H. vulgare*).

### 3.14. Protein-Protein Interaction Network Prediction of HvlecRLKs

**Figure 13:**
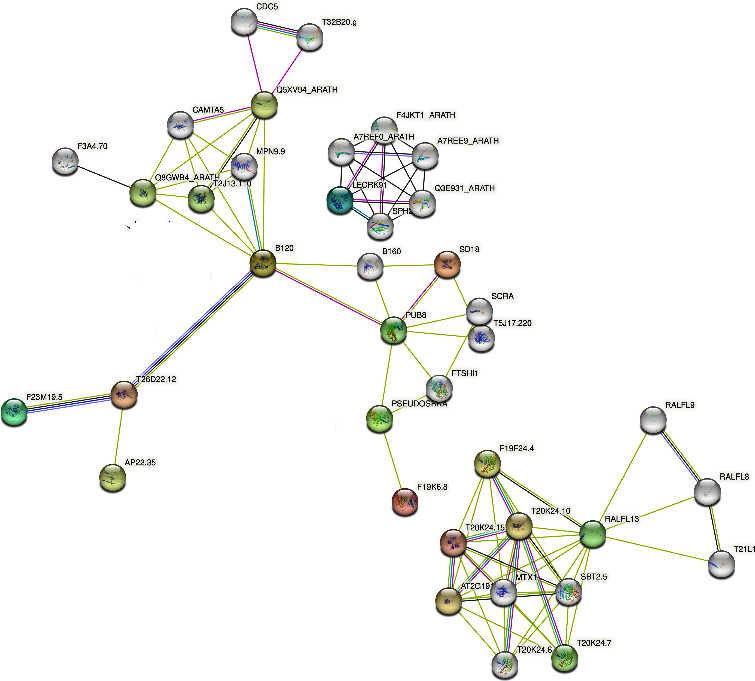
The protein-protein interaction network of HvlecRLK proteins. The proteins are represented at network nodes and the colored lines indicate different data sources. The thicker interaction lines between proteins indicate the higher coefficient and vice versa.

The protein-protein interaction was predicted between HvlecRLKs by STRING, based on the *Arabidopsis* (*A. thaliana*) orthologs to reveal their functions. For a specific gene family, protein-protein interaction networks provide valuable insight into the relationship with known protein family members [151]. Among all, 63 HvlecRLK proteins had a strong interaction with known *Arabidopsis* STRING proteins (Figure 13). In total, 29 HvlecRLK proteins were homologous with AtT20K24.15 and interacted with AtT20K24.6, AtT20K24.7, AtT20K24.10, AtF19F24.4, AT2G191, MTX1, RA2F13, and SBT25 and probably involved in kinase activity and metabolic process of plant species. Furthermore, 14 HvlecRLK proteins were homologous with AtB120 which highly interacted with AtB160, AtPUB8, AtT26D22.12, AtCAMTA5, AtQ5XV94_ARATH, AtMPN9.9, AtT2J13.110, and AtQ8GWB4_ARATH. AtB120 STRING protein was predicted to be involved in stress response and defense mechanisms [152]. Moreover, 9 HvlecRLKs were homologous with AtlecRLK91, linked to AtA7REF0_ARATH, AtQ3E931_ARATH, AtA7REE9_ARATH, AtF4JKT1_ARATH, and SPH2. HvleckRLK7, HvleckRLK9, HvleckRLK10, HvleckRLK20, HvleckRLK27, HvleckRLK34, HvleckRLK35, and HvleckRLK40 were also homologous to AtSD18 showing strong interaction with AtPUB8, AtB160, and AtSCRA. *Arabidopsis* STRING protein AtSD18 regulates plant pathogen interaction mediating bacterial lipopolysaccharide sensing [32]. HvleckRLK46, HvleckRLK42, and HvleckRLK19 were homologous with AtT26D22.12, AtF23M19.5, and AtPSEUDOSRKA, respectively. AtT26D22.12 interacted with AtB120, AtAP22.35, and AtF23M19.5 having strong catalytic activity. AtF23M19.5 proteins were highly connected to AtAP22.35 and AtT26D22.12 which may be involved in pollen recognition as well as cellular metabolic processes. AtPSEUDOSRKA was linked to AtF19K6.8, AtFTSHI1, and AtPUB8. AtPSEUDOSRKA was demonstrated as the key factor for determining self-incompatibility [21]. It has been previously proven that the interacted proteins function similarly [153]. Thus, HvlecRLK proteins which highly interacted with *Arabidopsis* known proteins might have similar functions.

## 4. Conclusion

In this study, we utilized the integrated bioinformatics approaches for the *in silico* identification and characterization of *LecRLK* genes in the barley genome (*H. vulgare* L.). A total of 113 *LecRLK* genes were identified and phylogenetically classified into three main categories (G-type, C-type, and L-type *HvlecRLK*) which maintain a close evolutionary relationship with *AtlecRLKs*. The predicted chromosomal location revealed that these *HvlecRLK* genes were unevenly distributed across 8 chromosomes including an unknown chromosome. The domain, motif, and exon-intron organization of *HvlecRLKs* demonstrated remarkable homogeneity with the corresponding gene family of *Arabidopsis*. The Ka/Ks ratios and collinear and syntenic gene pairs provide insight into the evolution of *HvlecRLK* genes. Furthermore, the GO analysis revealed the involvement of the identified *HvlecRLk* genes in several crucial biological, cellular, and molecular functions. The subcellular localization analysis identified the maximum protein signal in the plasma membrane indicating their involvement in the defense mechanism. The regulatory network and subnetwork analysis determined the presence of 29 TFFs including AP2, bZIP, C2H2, Dof, ERF, MIKC_MADS, MYB, NAC, and WRKY families linked to the putative *LecRLK* genes of barley. Furthermore, the cis-acting element analysis demonstrated the presence of CAREs in the *HvlecRLKs* promoter region associated with the response to light, tissue-specific, hormone, and stress. The predicted TFs were expected to bind with the CAREs of *HvlecRLKs* boosting plant growth and development as well as *LecRLK* gene expression of barley (*H. vulgare*). Thus, the findings might provide a strong basis for further functional investigation, characterization, and improvement of the *LecRLK* genes in wet lab experiments. This research has the potential to be valuable in breeding programs for this economically important cereal grain in the future.

## Figures and Tables

**Table 1:** List of 113 *LecRLK* genes of barley and their basic physiochemical characterization.

Gene ID	Gene name	Chromosomal location	ORF (bp)	Gene length (bp)	Intron	Protein		
						M.W (kD)	A.A	pI
HORVU0Hr1G014630	*HvleckRLK1*	chrUn:80417666..80425297	2526	7631	6	92.49	842	5.77
HORVU0Hr1G014650	*HvleckRLK2*	chrUn:80519134..80522430	2562	3296	6	81.51	854	8.01
HORVU1Hr1G001770	*HvleckRLK3*	chr1H:3879699..3888876	2511	9177	0	91.51	837	8.4
HORVU1Hr1G002000	*HvleckRLK4*	chr1H:4226239..4229021	2529	2782	0	93.03	843	8.32
HORVU1Hr1G002060	*HvleckRLK5*	chr1H:4269558..4277621	2625	8063	1	96.38	875	8.11
HORVU1Hr1G020020	*HvleckRLK6*	chr1H:77001064..77004795	2586	3731	4	96.23	862	7.97
HORVU1Hr1G066190	*HvleckRLK7*	chr1H:471017692..471025244	2571	7552	7	92.57	857	6.02
HORVU2Hr1G002830	*HvleckRLK8*	chr2H:6243900..6248436	2484	4536	0	90.98	828	7.01
HORVU2Hr1G008130	*HvleckRLK9*	chr2H:16822071..16828312	1755	6241	3	63.92	585	7.89
HORVU2Hr1G008140	*HvleckRLK10*	chr2H:16834473..16848850	2367	14377	5	87.32	789	6.45
HORVU2Hr1G042210	*HvleckRLK11*	chr2H:211334302..211509228	2466	174926	0	90.84	822	6.68
HORVU2Hr1G042220	*HvleckRLK12*	chr2H:211334342..211336901	2412	2559	0	88.9	804	7.53
HORVU2Hr1G042520	*HvleckRLK13*	chr2H:214213832..214218647	2595	4815	6	94	865	6.15
HORVU2Hr1G074430	*HvleckRLK14*	chr2H:537379810..537401427	2493	21617	0	90.96	831	6.24
HORVU2Hr1G074520	*HvleckRLK15*	chr2H:537565168..537568360	2424	3192	0	87.78	808	6.52
HORVU2Hr1G088570	*HvleckRLK16*	chr2H:633869191..634094741	2583	225550	0	95.6	861	7.92
HORVU2Hr1G108530	*HvleckRLK17*	chr2H:714614756..714618208	1518	3452	1	55.61	506	8.78
HORVU2Hr1G112090	*HvleckRLK18*	chr2H:724963768..724970047	2712	6279	1	99.11	904	8.17
HORVU2Hr1G117290	*HvleckRLK19*	chr2H:739993634..739998560	1575	4926	2	58.32	525	7.99
HORVU2Hr1G117360	*HvleckRLK20*	chr2H:740036862..740048150	2529	11288	6	92.06	843	5.93
HORVU2Hr1G117660	*HvleckRLK21*	chr2H:740674105..740677453	2439	3348	6	90.58	813	8.09
HORVU2Hr1G117670	*HvleckRLK22*	chr2H:740682512..740693131	2646	10619	6	97.97	882	6.31
HORVU2Hr1G117680	*HvleckRLK23*	chr2H:740694704..740698896	2379	4192	6	87.78	793	6.68
HORVU2Hr1G117790	*HvleckRLK24*	chr2H:740913556..740916778	2562	3222	5	95.55	854	6.28
HORVU2Hr1G117840	*HvleckRLK25*	chr2H:741026994..741037562	2442	10568	7	90.68	814	6.69
HORVU2Hr1G117870	*HvleckRLK26*	chr2H:741051084..741054878	2562	3794	5	95.55	854	6.28
HORVU2Hr1G121080	*HvleckRLK27*	chr2H:750744194..750750459	2598	6265	5	94.05	866	6.34
HORVU3Hr1G013180	*HvleckRLK28*	chr3H:28467930..28470566	2517	2636	0	92.76	839	7.08
HORVU3Hr1G013390	*HvleckRLK29*	chr3H:28939214..28942360	2487	3146	0	90.88	829	6.24
HORVU3Hr1G024650	*HvleckRLK30*	chr3H:96137708..96145501	2481	7793	2	89.11	827	6.37
HORVU3Hr1G030100	*HvleckRLK31*	chr3H:141730356..141738092	2427	7736	0	87.61	809	6.07
HORVU3Hr1G077110	*HvleckRLK32*	chr3H:571493241..571499445	2439	6204	6	89.76	813	5.97
HORVU3Hr1G077130	*HvleckRLK33*	chr3H:571554416..571565073	2493	10657	6	92.03	831	6.08
HORVU3Hr1G077170	*HvleckRLK34*	chr3H:571729834..571805674	2736	75840	5	100.16	912	8.62
HORVU3Hr1G077220	*HvleckRLK35*	chr3H:571947090..571954431	1863	7341	4	66.67	621	8.12
HORVU3Hr1G090180	*HvleckRLK36*	chr3H:630880155..630883691	2463	3536	1	88.94	821	6.35
HORVU4Hr1G067140	*HvleckRLK37*	chr4H:557588758..557591818	2553	3060	0	92.3	851	7.33
HORVU5Hr1G000240	*HvleckRLK38*	chr5H:1046549..1051684	927	5135	0	32.4	309	5.31
HORVU5Hr1G004160	*HvleckRLK39*	chr5H:7863131..7866743	2532	3612	0	92.8	844	7.45
HORVU5Hr1G087040	*HvleckRLK40*	chr5H:577658365..577662765	1857	4400	3	63.75	619	6.16
HORVU5Hr1G104610	*HvleckRLK41*	chr5H:619697868..619700626	2598	2758	0	96.35	866	7.75
HORVU5Hr1G118460	*HvleckRLK42*	chr5H:652964038..652967879	2595	3841	0	92.15	865	7.11
HORVU5Hr1G124170	*HvleckRLK43*	chr5H:665428810..665439194	2151	10384	6	77.41	717	8.39
HORVU6Hr1G001580	*HvleckRLK44*	chr6H:4886974..4890292	2508	3318	4	92.87	836	6.85
HORVU6Hr1G032410	*HvleckRLK45*	chr6H:141985317..141988391	2598	3074	0	94.02	866	6.3
HORVU6Hr1G080460	*HvleckRLK46*	chr6H:541820252..541823529	2688	3277	0	94.67	896	6.49
HORVU6Hr1G090780	*HvleckRLK47*	chr6H:573550582..573560428	2454	9846	7	90.57	9846	7.43
HORVU6Hr1G090830	*HvleckRLK48*	chr6H:573638671..573660137	2439	21466	6	90.54	813	6.52
HORVU6Hr1G090870	*HvleckRLK49*	chr6H:573646936..573651829	2499	4893	6	92.38	833	8.07
HORVU7Hr1G031210	*HvleckRLK50*	chr7H:63125769..63149891	2469	24122	6	90.28	823	6.12
HORVU7Hr1G047150	*HvleckRLK51*	chr7H:156899228..156903776	2607	4548	1	95.08	869	6.33
HORVU7Hr1G089080	*HvleckRLK52*	chr7H:540510582..540516881	2433	6299	0	90.24	811	5.32
HORVU7Hr1G091140	*HvleckRLK53*	chr7H:556041359..556044066	2487	2707	0	90.47	829	6.23
HORVU7Hr1G098630	*HvleckRLK54*	chr7H:598959299..598962247	2484	2948	0	91.16	828	8.07
HORVU7Hr1G098950	*HvleckRLK55*	chr7H:599392596..599397256	2409	4660	0	88.45	803	7.16
HORVU7Hr1G098960	*HvleckRLK56*	chr7H:599407352..599424432	2082	17080	0	76.61	694	8.8
HORVU7Hr1G099030	*HvleckRLK57*	chr7H:599463250..599467834	2571	4584	0	94.73	857	8.38
HORVU7Hr1G101700	*HvleckRLK58*	chr7H:610216440..610219730	2466	3290	0	89.71	822	8.19
HORVU7Hr1G105150	*HvleckRLK59*	chr7H:616214322..616217218	2454	2896	0	89.48	818	5.69
HORVU7Hr1G105170	*HvleckRLK60*	chr7H:616251556..616254571	2445	3015	0	88.28	815	6.48
HORVU7Hr1G105190	*HvleckRLK61*	chr7H:616319384..616322558	2433	3174	1	86.18	811	7.43
HORVU7Hr1G109340	*HvleckRLK62*	chr7H:627715566..627719295	2430	3729	6	89.37	810	7.13
HORVU3Hr1G014230	*HvleckRLK63*	chr3H:32573759..32577941	1845	4182	3	67.7	615	9.34
HORVU0Hr1G020280	*HvleckRLK64*	chrUn:106570325..106572493	1635	2168	0	58.76	545	7.17
HORVU0Hr1G022290	*HvleckRLK65*	chrUn:114437771..114440172	2085	2401	0	76.59	695	8.47
HORVU1Hr1G009250	*HvleckRLK66*	chr1H:20470978..20491157	1851	20179	0	68.98	617	6.58
HORVU1Hr1G013700	*HvleckRLK67*	chr1H:36739108..36743669	1215	4561	0	41.26	405	6.43
HORVU1Hr1G036970	*HvleckRLK68*	chr1H:251165013..251168604	2025	3591	0	73.44	675	6.75
HORVU1Hr1G037000	*HvleckRLK69*	chr1H:251208505..251259955	1998	51450	0	71.81	666	6.27
HORVU1Hr1G070040	*HvleckRLK70*	chr1H:487995237..487999364	2055	4127	0	73.45	685	9.14
HORVU2Hr1G006100	*HvleckRLK71*	chr2H:13004115..13006667	1959	2552	0	71.71	653	6.17
HORVU2Hr1G014890	*HvleckRLK72*	chr2H:32696272..32706701	2073	10429	0	75.74	691	8.32
HORVU2Hr1G014900	*HvleckRLK73*	chr2H:32711201..33211836	2070	500635	0	76.09	690	6.42
HORVU2Hr1G014930	*HvleckRLK74*	chr2H:32743275..32745801	2232	2526	0	82.24	744	7.03
HORVU2Hr1G037200	*HvleckRLK75*	chr2H:168843396..168845766	2076	2370	0	76.01	692	6.73
HORVU2Hr1G037210	*HvleckRLK76*	chr2H:168860173..168862521	2040	2348	0	74.54	680	7.08
HORVU2Hr1G038790	*HvleckRLK77*	chr2H:183865291..183867373	2082	2082	0	77.38	694	6.95
HORVU2Hr1G091360	*HvleckRLK78*	chr2H:647828572..647831604	2031	3032	0	73.6	677	6.58
HORVU2Hr1G104610	*HvleckRLK79*	chr2H:704172005..704174498	2163	2493	0	80.15	721	9.12
HORVU2Hr1G120660	*HvleckRLK80*	chr2H:749760054..749824545	1953	64491	0	72.49	651	6.92
HORVU2Hr1G125230	*HvleckRLK81*	chr7H:156899228..156903776	2607	4548	1	95.08	869	6.33
HORVU3Hr1G015210	*HvleckRLK82*	chr3H:35556561..35558304	1743	1743	0	64.53	581	6.76
HORVU3Hr1G018500	*HvleckRLK83*	chr3H:48455621..48457658	2037	2037	0	74.1	679	6.33
HORVU3Hr1G018610	*HvleckRLK84*	chr3H:48628065..48632120	2022	4055	0	73.58	674	6.23
HORVU3Hr1G018690	*HvleckRLK85*	chr3H:48685871..48688520	2031	2649	0	74.99	677	6.52
HORVU3Hr1G059850	*HvleckRLK86*	chr3H:455125231..455127725	2163	2494	1	77.43	721	5.4
HORVU3Hr1G076680	*HvleckRLK87*	chr3H:569417182..569429535	2088	12353	0	75.6	696	6.23
HORVU4Hr1G016880	*HvleckRLK88*	chr4H:71109936..71112798	2163	2862	1	77.43	721	5.4
HORVU4Hr1G075550	*HvleckRLK89*	chr4H:598754028..598757520	2013	3492	0	72.12	671	6.12
HORVU5Hr1G000940	*HvleckRLK90*	chr5H:3324589..3328788	2148	4199	3	79.24	716	5.92
HORVU5Hr1G020530	*HvleckRLK91*	chr5H:95189966..95192675	2130	2709	0	76.03	710	7.86
HORVU5Hr1G098640	*HvleckRLK92*	chr5H:608357746..608360809	2055	3063	0	74.58	685	6.51
HORVU5Hr1G104840	*HvleckRLK93*	chr5H:620162907..620165410	2067	2503	0	75.21	689	7.47
HORVU5Hr1G104850	*HvleckRLK94*	chr5H:620173770..620176278	1788	2508	0	65.37	596	9.07
HORVU5Hr1G110920	*HvleckRLK95*	chr5H:634496326..634512632	1617	16306	0	59.77	539	6.77
HORVU5Hr1G114030	*HvleckRLK96*	chr5H:643015642..643017912	2169	2270	0	77.57	723	6.07
HORVU5Hr1G114100	*HvleckRLK97*	chr5H:643118584..643121019	2124	2435	0	76.99	708	5.88
HORVU6Hr1G025340	*HvleckRLK98*	chr6H:91923166..91926226	2139	3060	1	77.88	713	6.01
HORVU6Hr1G025350	*HvleckRLK99*	chr6H:91936047..91938968	2055	2921	0	74.74	685	6.92
HORVU6Hr1G053090	*HvleckRLK100*	chr6H:328630263..328632697	2127	2434	0	78.24	709	7.33
HORVU6Hr1G053120	*HvleckRLK101*	chr6H:328938303..328940712	2007	2409	0	73.72	669	7.78
HORVU6Hr1G060540	*HvleckRLK102*	chr6H:402637101..402639578	2013	2477	0	73.35	671	6.24
HORVU6Hr1G069980	*HvleckRLK103*	chr6H:485989955..485993554	2373	3599	1	84.87	791	7.58
HORVU6Hr1G084370	*HvleckRLK104*	chr6H:554176455..554178973	2064	2518	0	76.17	688	6.86
HORVU6Hr1G093300	*HvleckRLK105*	chr6H:579277929..579296627	2016	18698	0	73.99	672	6.11
HORVU7Hr1G000530	*HvleckRLK106*	chr7H:786240..788617	2061	2377	0	75.24	687	6.91
HORVU7Hr1G000830	*HvleckRLK107*	chr7H:1767721..1769985	2088	2264	0	76	696	6.22
HORVU7Hr1G019390	*HvleckRLK108*	chr7H:25786151..25788866	2121	2715	0	77.58	707	6.8
HORVU7Hr1G019400	*HvleckRLK109*	chr7H:25818141..25820583	2022	2442	0	73.82	674	6.71
HORVU7Hr1G028730	*HvleckRLK110*	chr7H:53030575..53036745	2067	6170	0	76.33	689	5.67
HORVU7Hr1G043490	*HvleckRLK111*	chr7H:131263083..131265641	2406	2558	0	85.27	802	8.64
HORVU7Hr1G074760	*HvleckRLK112*	chr7H:429080348..429085289	1494	4941	1	54.58	498	7.26
HORVU7Hr1G098030	*HvleckRLK113*	chr7H:594740884..594742966	2082	2082	0	77.39	694	6.95

**Table 2:** Information about abundant miRNA ID, functions, and their targeted *HvlecRLK* genes.

miRNA ID	Functions	Targeted genes^a^	References
hvu-miR6204	Target the genes of SAUR-like auxin-responsive protein family, responsible for auxin metabolism	13, 36, 45, 46, 58, 68, 78, 86, 88, 89, 91, 92, 93, 94, 96, 99, 100, 105, 109	[139]
hvu-miR6214	Implicated in inducing stress responses as well as antioxidant system	2, 7, 15, 34, 37, 42, 44, 66, 69, 78, 87, 90, 92, 96, 97, 101, 102	[140]
hvu-miR6196	Play a pivotal role in salt stress treatment being unregulated in diploid stress	6, 9, 13, 14, 34, 46, 63, 71, 72, 82, 83, 84, 89, 103, 106, 111	[141]
hvu-miR169	Differentially expressed under potassium (*K*) stress regulating various photosynthetic processes; involved in plant stress tolerance in various nitrogen (*N*) levels	6, 8, 10, 11, 27, 55, 63, 66, 73, 87, 92, 108	[142, 143]

*Note*. ^a^Supplementary Table 1.

## Data Availability

The datasets used and/or analyzed during the current study are available from the corresponding author upon reasonable request by e-mail.

## Supplementary Materials

Supplementary Materials S1 Data: protein sequences of HvlecRLKs (txt). S2 Data: CDS sequences of HvlecRLKs (txt). S3 Data: genomic sequences of HvlecRLKs (txt). Supplementary Table 1: miRNA targeted HvlecRLKs (Doc).
