# Supplementary material for: Genome-Wide Comprehensive Identification and In Silico Characterization of Lectin Receptor-Like Kinase Gene Family in Barley (Hordeum vulgare L.)
Source: Genet Res (Camb). 2024 Feb 27;2024:2924953. doi: 10.1155/2024/2924953 (PMC10914435; doi:10.1155/2024/2924953)
Supplement: Supplementary Materials — S1 Data: protein sequences of HvlecRLKs (txt). S2 Data: CDS sequences of HvlecRLKs (txt). S3 Data: genomic sequences of HvlecRLKs (txt). Supplementary Table 1: miRNA targeted HvlecRLKs (Doc). [file 2924953.f1.zip › Supplementary Table 1.miRNA targeted HvlecRLKs.pdf]

Supplementary Table 1. miRNA targeted prediction of *HvlecRLK*s. The miRNA data was downloaded from the plant micro-RNA encyclopedia (http://pmiren.com/).

miRNA ID	Target ID	Target length	Target start	Target end	miRNA_aligned_fragment
hvu-miR6204	*HvleckRLK1*	22	29	50	AGGAGAAUAAUUAGAGCUGUGA
hvu-miR5048a	*HvleckRLK1*	22	1945	1966	UAUUUGCAGGUUUUAGGUCUAA
hvu-miR5048b	*HvleckRLK1*	22	1945	1966	UAUUUGCAGGUUUUAGGUCUAA
hvu-miR6195	*HvleckRLK1*	21	2206	2226	UGAGUACGUAGUAGGGAUGAG
hvu-miR6192	*HvleckRLK10*	22	27	48	UAGGAGAGGGGGGAAGGGAUCU
hvu-miR6214	*HvleckRLK100*	20	408	427	CGACGACGACGAGCACGACA
hvu-miR169	*HvleckRLK101*	21	762	782	AAGCCAAGGAUGAGUUGCCUG
hvu-miR6184	*HvleckRLK101*	22	1190	1211	CGGCGUCGGAUCUGGCCGGCCU
hvu-miR6184	*HvleckRLK102*	22	1193	1214	CGGCGUCGGAUCUGGCCGGCCU
hvu-miR6198	*HvleckRLK102*	22	701	722	GCUCUGUCUUGGAUGGUCAUUC
hvu-miR6196	*HvleckRLK103*	21	299	319	AGGACGAGGAGAUGGAGAGGA
hvu-miR6203	*HvleckRLK104*	22	1720	1741	AGACGAUUAAGAAGACCUGCAA
hvu-miR6208	*HvleckRLK104*	22	1293	1314	GUGCAUCAAGAUCGGCUCAUCU
hvu-miR6214	*HvleckRLK105*	20	24	43	CGACGACGACGAGCACGACA
hvu-miR6196	*HvleckRLK106*	21	263	284	AGGACG-AGGAGAUGGAGAGGA
hvu-miR6208	*HvleckRLK107*	22	655	676	GUGCAUCAAGAUCGGCUCAUCU
hvu-miR168-5p	*HvleckRLK108*	21	1121	1141	UCGCUUGGUGCAGAUCGGGAC
hvu-miR6192	*HvleckRLK108*	22	7	28	UAGGAGAGGGGGGAAGGGAUCU
hvu-miR6214	*HvleckRLK109*	20	717	738	CGACGACGACGAGCA--CGACA
hvu-miR6192	*HvleckRLK11*	22	3	24	UAGGAGAGGGGGGAAGGGAUCU
hvu-miR159a	*HvleckRLK11*	21	2366	2386	UUUGGAUUGAAGGGAGCUCUG
hvu-miR159b	*HvleckRLK11*	21	2366	2386	UUUGGAUUGAAGGGAGCUCUG
hvu-miR6196	*HvleckRLK111*	21	677	697	AGGACGAGGAGAUGGAGAGGA
hvu-miR159a	*HvleckRLK12*	21	2312	2332	UUUGGAUUGAAGGGAGCUCUG
hvu-miR159b	*HvleckRLK12*	21	2312	2332	UUUGGAUUGAAGGGAGCUCUG
hvu-miR6196	*HvleckRLK13*	21	143	163	AGGACGAGGAGAUGGAGAGGA
hvu-miR6198	*HvleckRLK13*	22	374	395	GCUCUGUCUUGGAUGGUCAUUC
hvu-miR6214	*HvleckRLK13*	20	2333	2352	CGACGACGACGAGCACGACA
hvu-miR168-5p	*HvleckRLK13*	21	1036	1056	UCGCUUGGUGCAGAUCGGGAC
hvu-miR5053	*HvleckRLK13*	21	941	961	CGCAGCUGUAGUCGCCGGCGU
hvu-miR6181	*HvleckRLK13*	23	1011	1033	UGCUCUUCAUGGACUGCGGCGCC
hvu-miR6187	*HvleckRLK13*	21	704	724	UGAACAGGUUCGGCGACCUCA
hvu-miR6189	*HvleckRLK13*	20	257	276	AGGUGAUGCUGUGGUGAUCU
hvu-miR6196	*HvleckRLK14*	21	87	107	AGGACGAGGAGAUGGAGAGGA
hvu-miR159a	*HvleckRLK14*	21	1829	1849	UUUGGAUUGAAGGGAGCUCUG
hvu-miR159b	*HvleckRLK14*	21	1829	1849	UUUGGAUUGAAGGGAGCUCUG
hvu-miR6179	*HvleckRLK14*	22	59	80	AACCAGUCGAGGCCAGGGGGUU
hvu-miR6184	*HvleckRLK15*	22	524	545	CGGCGUCGGAUCUGGCCGGCCU

Supplementary Table 1 (Continued)

miRNA ID	Target ID	Target length	Target start	Target end	miRNA_aligned_fragment
hvu-miR6189	*HvleckRLK18*	20	1977	1995	AGGUGAUGCUGUGGUGAUCU
hvu-miR6184	*HvleckRLK2*	22	322	343	CGGCGUCGGAUCUGGCCGGCCU
hvu-miR5051	*HvleckRLK2*	21	1089	1109	UUUGGCACCUUGAAACUGGGA
hvu-miR6189	*HvleckRLK20*	20	123	142	AGGUGAUGCUGUGGUGAUCU
hvu-miR397b-3p	*HvleckRLK21*	21	1060	1080	AUCAACGCUGCACUCAACGGC
hvu-miR6195	*HvleckRLK23*	21	1239	1258	UGAGUACGUAGUAGGGAUGAG
hvu-miR5053	*HvleckRLK27*	21	1178	1198	CGCAGCUGUAGUCGCCGGCGU
hvu-miR5051	*HvleckRLK27*	21	2217	2237	UUUGGCACCUUGAAACUGGGA
hvu-miR6192	*HvleckRLK27*	22	31	51	UAGGAGAGGGGGGAAGGGAUCU
hvu-miR6191	*HvleckRLK28*	20	901	920	UAGAUUUGUCUAGAUAUGAA
hvu-miR166a	*HvleckRLK31*	21	1784	1804	UCGGACCAGGCUUCAUUCCCC
hvu-miR166b	*HvleckRLK31*	21	1784	1804	UCGGACCAGGCUUCAUUCCCC
hvu-miR166c	*HvleckRLK31*	21	1784	1804	UCGGACCAGGCUUCAUUCCCC
hvu-miR6207	*HvleckRLK31*	21	8	28	UGGACGACCUGGGCGCCGACG
hvu-miR6202	*HvleckRLK32*	20	159	178	UGAAGAUUUUAAGCAUUGAA
hvu-miR1120	*HvleckRLK32*	24	1599	1622	ACAUUCUUAUAUUAUGGGACGGAG
hvu-miR1130	*HvleckRLK33*	21	1379	1399	UCUGUAACUUAAUAUAAGACG
hvu-miR6202	*HvleckRLK33*	20	162	181	UGAAGAUUUUAAGCAUUGAA
hvu-miR6196	*HvleckRLK34*	21	224	244	AGGACGAGGAGAUGGAGAGGA
hvu-miR444b	*HvleckRLK34*	21	1052	1072	UGCAGUUGCUGUCUCAAGCUU
hvu-miR6182	*HvleckRLK34*	22	53	74	UGAGUGUGUGAUGGAUGGCUUU
hvu-miR6184	*HvleckRLK34*	22	337	358	CGGCGUCGGAUCUGGCCGGCCU
hvu-miR5053	*HvleckRLK35*	21	1193	1213	CGCAGCUGUAGUCGCCGGCGU
hvu-miR6214	*HvleckRLK36*	20	1383	1402	CGACGACGACGAGCACGACA
hvu-miR171-5p	*HvleckRLK36*	21	846	867	UGUUGGCUCGACUCAC-UCAGA
hvu-miR6184	*HvleckRLK37*	22	881	902	CGGCGUCGGAUCUGGCCGGCCU
hvu-miR6207	*HvleckRLK37*	21	623	643	UGGACGACCUGGGCGCCGACG
hvu-miR5048a	*HvleckRLK39*	22	1185	1206	UAUUUGCAGGUUUUAGGUCUAA
hvu-miR5048b	*HvleckRLK39*	22	1185	1206	UAUUUGCAGGUUUUAGGUCUAA
hvu-miR6206	*HvleckRLK39*	22	954	975	GGCACACGGGCUGCAGGCAUAG
hvu-miR6193	*HvleckRLK4*	23	507	530	CUCUGCCACCGGUC-CAUGACGAC
hvu-miR6184	*HvleckRLK42*	22	1517	1537	CGGCGUCGGAUCUGGCCGGCCU
hvu-miR6207	*HvleckRLK42*	21	1314	1334	UGGACGACCUGGGCGCCGACG
hvu-miR6207	*HvleckRLK42*	21	323	343	UGGACGACCUGGGCGCCGACG
hvu-miR5051	*HvleckRLK43*	21	678	698	UUUGGCACCUUGAAACUGGGA
hvu-miR169	*HvleckRLK44*	21	425	445	AAGCCAAGGAUGAGUUGCCUG
hvu-miR6184	*HvleckRLK44*	22	2092	2113	CGGCGUCGGAUCUGGCCGGCCU
hvu-miR6188	*HvleckRLK44*	22	2234	2255	GGUGGAUCGAUGAACCCGGCGA
hvu-miR6214	*HvleckRLK45*	20	447	465	CGACGACGACGAGCACGACA
hvu-miR6214	*HvleckRLK46*	20	1368	1387	CGACGACGACGAGCACGACA

Supplementary Table 1 (Continued)

miRNA ID	Target ID	Target length	Target start	Target end	miRNA_aligned_fragment
hvu-miR6214	*HvleckRLK46*	20	815	834	CGACGACGACGAGCACGACA
hvu-miR5052	*HvleckRLK46*	21	759	779	ACCGGCUGGACGGUAGGCAUA
hvu-miR6214	*HvleckRLK46*	20	2648	2667	CGACGACGACGAGCACGACA
hvu-miR6196	*HvleckRLK46*	21	1178	1198	AGGACGAGGAGAUGGAGAGGA
hvu-miR5048a	*HvleckRLK5*	22	1272	1293	UAUUUGCAGGUUUUAGGUCUAA
hvu-miR5048b	*HvleckRLK5*	22	1272	1293	UAUUUGCAGGUUUUAGGUCUAA
hvu-miR169	*HvleckRLK5*	21	224	245	AAGCCAAGGAUGAG-UUGCCUG
hvu-miR6198	*HvleckRLK50*	22	2370	2391	GCUCUGUCUUGGAUGGUCAUUC
hvu-miR6210	*HvleckRLK51*	22	2071	2092	ACUCCUUGGUUAUCAACUUCGA
hvu-miR6202	*HvleckRLK52*	20	304	323	UGAAGAUUUUAAGCAUUGAA
hvu-miR5053	*HvleckRLK54*	21	2123	2143	CGCAGCUGUAGUCGCCGGCGU
hvu-miR159a	*HvleckRLK55*	21	696	716	UUUGGAUUGAAGGGAGCUCUG
hvu-miR159b	*HvleckRLK55*	21	696	716	UUUGGAUUGAAGGGAGCUCUG
hvu-miR6192	*HvleckRLK55*	22	1279	1300	UAGGAGAGGGGGGAAGGGAUCU
hvu-miR6206	*HvleckRLK57*	22	89	110	GGCACACGGGCUGCAGGCAUAG
hvu-miR5053	*HvleckRLK58*	21	1562	1582	CGCAGCUGUAGUCGCCGGCGU
hvu-miR6194	*HvleckRLK58*	21	1280	1300	UAUGGGGAUCUGACAGACGAG
hvu-miR6214	*HvleckRLK58*	20	365	384	CGACGACGACGAGCACGACA
hvu-miR6185	*HvleckRLK59*	21	1071	1091	UCUGGCAGCGACGGGAACAUA
hvu-miR6196	*HvleckRLK6*	21	117	137	AGGACGAGGAGAUGGAGAGGA
hvu-miR6188	*HvleckRLK6*	22	2318	2339	GGUGGAUCGAUGAACCCGGCGA
hvu-miR6192	*HvleckRLK6*	22	117	138	UAGGAGAGGGGGGAAGGGAUCU
hvu-miR6182	*HvleckRLK60*	22	772	792	UGAGUGUGUGAUGGAUGGCUUU
hvu-miR5053	*HvleckRLK60*	21	2081	2101	CGCAGCUGUAGUCGCCGGCGU
hvu-miR6185	*HvleckRLK60*	21	357	377	UCUGGCAGCGACGGGAACAUA
hvu-miR444a	*HvleckRLK61*	21	2165	2185	UUGCUGCCUCAAGCUUGCUGC
hvu-miR5053	*HvleckRLK61*	21	1559	1579	CGCAGCUGUAGUCGCCGGCGU
hvu-miR6185	*HvleckRLK61*	21	1092	1112	UCUGGCAGCGACGGGAACAUA
hvu-miR6196	*HvleckRLK63*	21	43	63	AGGACGAGGAGAUGGAGAGGA
hvu-miR6192	*HvleckRLK63*	22	38	58	UAGGAGAGGGGGGAAGGGAUCU
hvu-miR6184	*HvleckRLK66*	22	1178	1199	CGGCGUCGGAUCUGGCCGGCCU
hvu-miR6192	*HvleckRLK66*	22	14	35	UAGGAGAGGGGGGAAGGGAUCU
hvu-miR6187	*HvleckRLK68*	21	350	370	UGAACAGGUUCGGCGACCUCA
hvu-miR6214	*HvleckRLK68*	20	723	741	CGACGACGACGAGCACGACA
hvu-miR6214	*HvleckRLK68*	20	906	925	CGACGACGACGAGCACGACA
hvu-miR6184	*HvleckRLK69*	22	1154	1175	CGGCGUCGGAUCUGGCCGGCCU
hvu-miR6208	*HvleckRLK7*	22	1291	1312	GUGCAUCAAGAUCGGCUCAUCU
hvu-miR6184	*HvleckRLK7*	22	112	133	CGGCGUCGGAUCUGGCCGGCCU
hvu-miR6196	*HvleckRLK71*	21	200	220	AGGACGAGGAGAUGGAGAGGA
hvu-miR6196	*HvleckRLK72*	21	349	370	AGGACGAGGAGAUG-GAGAGGA

Supplementary Table 1 (Continued)

miRNA ID	Target ID	Target length	Target start	Target end	miRNA_aligned_fragment
hvu-miR169	*HvleckRLK72*	21	783	803	AAGCCAAGGAUGAGUUGCCUG
hvu-miR6194	*HvleckRLK72*	21	741	761	UAUGGGGAUCUGACAGACGAG
hvu-miR6192	*HvleckRLK73*	22	210	231	UAGGAGAGGGGGGAAGGGAUCU
hvu-miR169	*HvleckRLK74*	21	957	977	AAGCCAAGGAUGAGUUGCCUG
hvu-miR5052	*HvleckRLK74*	21	374	395	ACCGGCUGGACGGUA-GGCAUA
hvu-miR6193	*HvleckRLK74*	23	343	367	CUCUGCCACCGGUC--CAUGACGAC
hvu-miR6184	*HvleckRLK78*	22	1193	1214	CGGCGUCGGAUCUGGCCGGCCU
hvu-miR6214	*HvleckRLK78*	20	726	744	CGACGACGACGAGCACGACA
hvu-miR6176	*HvleckRLK79*	23	2105	2127	GAAGCUGUAGUGCAGCCGGCGUU
hvu-miR6207	*HvleckRLK79*	21	296	316	UGGACGACCUGGGCGCCGACG
hvu-miR6198	*HvleckRLK79*	22	304	325	GCUCUGUCUUGGAUGGUCAUUC
hvu-miR6192	*HvleckRLK8*	22	23	44	UAGGAGAGGGGGGAAGGGAUCU
hvu-miR1130	*HvleckRLK8*	21	1852	1872	UCUGUAACUUAAUAUAAGACG
hvu-miR399	*HvleckRLK81*	21	755	775	UGCCAAAGGAGAUUUGCCCCG
hvu-miR6196	*HvleckRLK82*	21	14	34	AGGACGAGGAGAUGGAGAGGA
hvu-miR159a	*HvleckRLK83*	21	1663	1682	UUUGGAUUGAAGGGAGCUCUG
hvu-miR159b	*HvleckRLK83*	21	1663	1682	UUUGGAUUGAAGGGAGCUCUG
hvu-miR168-5p	*HvleckRLK83*	21	1037	1057	UCGCUUGGUGCAGAUCGGGAC
hvu-miR6196	*HvleckRLK83*	21	33	53	AGGACGAGGAGAUGGAGAGGA
hvu-miR159a	*HvleckRLK84*	21	1648	1667	UUUGGAUUGAAGGGAGCUCUG
hvu-miR159b	*HvleckRLK84*	21	1648	1667	UUUGGAUUGAAGGGAGCUCUG
hvu-miR168-5p	*HvleckRLK84*	21	1022	1042	UCGCUUGGUGCAGAUCGGGAC
hvu-miR6196	*HvleckRLK84*	21	18	38	AGGACGAGGAGAUGGAGAGGA
hvu-miR6214	*HvleckRLK86*	20	924	943	CGACGACGACGAGCACGACA
hvu-miR6181	*HvleckRLK86*	23	1905	1926	UGCUCUUCAUGGACUGCGGCGCC
hvu-miR6193	*HvleckRLK86*	23	1294	1316	CUCUGCCACCGGUCCAUGACGAC
hvu-miR168-3p	*HvleckRLK87*	24	1100	1122	GAUCCCGCCUUGCACCAAGUGAAU
hvu-miR6184	*HvleckRLK87*	22	1108	1129	CGGCGUCGGAUCUGGCCGGCCU
hvu-miR6190	*HvleckRLK87*	21	406	426	CGAGGAAAGGAAGAAGCCAUG
hvu-miR6192	*HvleckRLK87*	22	341	362	UAGGAGAGGGGGGAAGGGAUCU
hvu-miR6214	*HvleckRLK88*	20	924	943	CGACGACGACGAGCACGACA
hvu-miR6181	*HvleckRLK88*	23	1905	1926	UGCUCUUCAUGGACUGCGGCGCC
hvu-miR6193	*HvleckRLK88*	23	1294	1316	CUCUGCCACCGGUCCAUGACGAC
hvu-miR6214	*HvleckRLK89*	20	36	55	CGACGACGACGAGCACGACA
hvu-miR6196	*HvleckRLK89*	21	1946	1966	AGGACGAGGAGAUGGAGAGGA
hvu-miR6196	*HvleckRLK9*	21	188	208	AGGACGAGGAGAUGGAGAGGA
hvu-miR6184	*HvleckRLK90*	22	634	655	CGGCGUCGGAUCUGGCCGGCCU
hvu-miR5052	*HvleckRLK90*	21	1871	1891	ACCGGCUGGACGGUAGGCAUA
hvu-miR6198	*HvleckRLK90*	22	687	708	GCUCUGUCUUGGAUGGUCAUUC
hvu-miR6214	*HvleckRLK91*	20	2003	2022	CGACGACGACGAGCACGACA

Supplementary Table 1 (Continued)

miRNA ID	Target ID	Target length	Target start	Target end	miRNA_aligned_fragment
hvu-miR6192	*HvleckRLK91*	22	60	81	UAGGAGAGGGGGGAAGGGAUCU
hvu-miR6189	*HvleckRLK91*	20	549	568	AGGUGAUGCUGUGGUGAUCU
hvu-miR6207	*HvleckRLK91*	21	618	638	UGGACGACCUGGGCGCCGACG
hvu-miR6184	*HvleckRLK92*	22	1205	1226	CGGCGUCGGAUCUGGCCGGCCU
hvu-miR6214	*HvleckRLK92*	20	921	940	CGACGACGACGAGCACGACA
hvu-miR6186	*HvleckRLK93*	21	1640	1660	CGAGGAAGGCGCUGAGAGAGA
hvu-miR6214	*HvleckRLK93*	20	765	783	CGACGACGACGAGCACGACA
hvu-miR6198	*HvleckRLK94*	22	1343	1364	GCUCUGUCUUGGAUGGUCAUUC
hvu-miR6214	*HvleckRLK94*	20	297	316	CGACGACGACGAGCACGACA
hvu-miR6195	*HvleckRLK95*	21	337	357	UGAGUACGUAGUAGGGAUGAG
hvu-miR6190	*HvleckRLK96*	21	113	133	CGAGGAAAGGAAGAAGCCAUG
hvu-miR168-5p	*HvleckRLK96*	21	269	289	UCGCUUGGUGCAGAUCGGGAC
hvu-miR5053	*HvleckRLK96*	21	1178	1198	CGCAGCUGUAGUCGCCGGCGU
hvu-miR6184	*HvleckRLK96*	22	1212	1231	CGGCGUCGGAUCUGGCCGGCCU
hvu-miR6185	*HvleckRLK96*	21	2076	2096	UCUGGCAGCGACGGGAACAUA
hvu-miR6207	*HvleckRLK96*	21	1449	1469	UGGACGACCUGGGCGCCGACG
hvu-miR6214	*HvleckRLK96*	20	2075	2094	CGACGACGACGAGCACGACA
hvu-miR6184	*HvleckRLK97*	22	1132	1153	CGGCGUCGGAUCUGGCCGGCCU
hvu-miR6208	*HvleckRLK99*	22	1281	1302	GUGCAUCAAGAUCGGCUCAUCU
hvu-miR6214	*HvleckRLK99*	20	939	958	CGACGACGACGAGCACGACA
hvu-miR6214	*HvleckRLK99*	20	759	777	CGACGACGACGAGCACGACA
